# Transcriptional landscapes of emerging autoimmunity: transient aberrations in the targeted tissue’s extracellular milieu precede immune responses in Sjögren’s syndrome

**DOI:** 10.1186/ar4362

**Published:** 2013-10-31

**Authors:** Nicolas Delaleu, Cuong Q Nguyen, Kidane M Tekle, Roland Jonsson, Ammon B Peck

**Affiliations:** 1 Broegelmann Research Laboratory, Department of Clinical Science, University of Bergen, Laboratory Building, Jonas Liesvei 65, 5021 Bergen, Norway; 2 Department of Infectious Diseases and Pathology, College of Veterinary Medicine, University of Florida, 2015 S.W. 16th Avenue, Gainesville, FL 32610, USA; 3 Computational Biology Unit, Uni-Computing, UniResearch AS, Thormøhlensgate 55, 5008 Bergen, Norway; 4 Department of Rheumatology, Haukeland University Hospital, Jonas Liesvei 65, 5021 Bergen, Norway

## Abstract

**Introduction:**

Our understanding of autoimmunity is skewed considerably towards the late stages of overt disease and chronic inflammation. Defining the targeted organ’s role during emergence of autoimmune diseases is, however, critical in order to define their etiology, early and covert disease phases and delineate their molecular basis.

**Methods:**

Using Sjögren’s syndrome (SS) as an exemplary rheumatic autoimmune disease and temporal global gene-expression profiling, we systematically mapped the transcriptional landscapes and chronological interrelationships between biological themes involving the salivary glands’ extracellular milieu. The time period studied spans from pre- to subclinical and ultimately to onset of overt disease in a well-defined model of spontaneous SS, the C57BL/6.NOD-*Aec1Aec2* strain. In order to answer this aim of great generality, we developed a novel bioinformatics-based approach, which integrates comprehensive data analysis and visualization within interactive networks. The latter are computed by projecting the datasets as a whole on *a priori*-defined consensus-based knowledge.

**Results:**

Applying these methodologies revealed extensive susceptibility loci-dependent aberrations in salivary gland homeostasis and integrity preceding onset of overt disease by a considerable amount of time. These alterations coincided with innate immune responses depending predominantly on genes located outside of the SS-predisposing loci *Aec1* and *Aec2*. Following a period of transcriptional stability, networks mapping the onset of overt SS displayed, in addition to natural killer, T- and B-cell-specific gene patterns, significant reversals of focal adhesion, cell-cell junctions and neurotransmitter receptor-associated alterations that had prior characterized progression from pre- to subclinical disease.

**Conclusions:**

This data-driven methodology advances unbiased assessment of global datasets an allowed comprehensive interpretation of complex alterations in biological states. Its application delineated a major involvement of the targeted organ during the emergence of experimental SS.

## Introduction

Common to autoimmune diseases is a long and clinically silent phase. As a consequence, affected individuals are diagnosed only after immune system–mediated functional deficiencies of the affected tissues result in overt disease [1,2]. Hence, owing to the unavailability of human specimens reflecting subclinical disease stages, understanding of the molecular basis of autoimmunity is skewed toward late and overt disease phases. To conclusively assign etiological relevance to any biological process altered in such specimens is difficult, considering the causality dilemma. Nevertheless, stratifying the chronology of these events is crucial in estimating whether genetic predisposition to develop a specific autoimmune disease might also involve genes associated with tissue development and homeostasis or if the genes exclusively cluster in processes associated with specific phases of innate adaptive immune maturation [3-5].

One approach to delineate, and to a certain extent stratify, the molecular events associated with subclinical phases of autoimmune diseases is the use of adequate experimental models [6,7]. For this purpose, a suitable experimental strain must, in correspondence with humans, develop its relevant autoimmune phenotype over an extended period of time and in the context of its genetic background. C57BL/6.NOD-*Aec1Aec2* mice fulfill these criteria as a model of primary Sjögren’s syndrome (SS) because they develop, in the absence of other inflammatory conditions, all major features relevant to the diagnosis of SS in humans spontaneously and over a period of several months [7,8].

With a prevalence of 0.1% to 0.3% in the total population, SS is considered a relatively common autoimmune disease. It mainly involves the exocrine glands. Nearly all patients complain about persistent symptoms of dry mouth, and many present with hyposalivation. Severe disease outcomes also include disabling fatigue and development of non-Hodgkin’s lymphoma. To date, all therapies tested have been ineffective in reversing the course of SS [9,10]. Similar to patients with systemic lupus erythematosus, a subpopulation of individuals with SS exhibit a type 1 IFN signature, suggesting that a viral agent may be involved in triggering the disease [11]. As a consequence, studies designed to discover genetic associations have focused either on innate immunity [12] or on genes that might explain the dominant role of B cells in the pathogenesis of SS [10]. Unfortunately, these studies have yet to yield results that allow estimation of an individual’s risk of developing SS.

Histological evaluations of minor salivary glands (SGs) obtained from patients with SS commonly show focal inflammation that may coincide with epithelial cell atrophy and the presence of adipose tissue and fibrosis. Morphologically, these glands may also display structural disorganization, including loss of cell–cell and cell–extracellular matrix (ECM) adhesion [13,14]. However, organizing these findings chronologically and conclusively as etiological, pathogenic or bystander processes has not yet been possible [9].

Thus, the aim of this study was to delineate the transcriptional landscape associated with the extracellular milieu (EM) of the SGs during spontaneous emergence of experimental SS. The global scope of our aim favors integration over reduction and is ideally based on a data-driven approach that ensures impartial interpretation of data sets as a whole. For this purpose, we developed a novel data analysis pipeline that combines gene set enrichment analyses (GSEAs) [15], leading edge (LE) analyses [15] and Markov cluster algorithm (MCL) clustering [16] for analysis of biological states. This set of data analyses formed the basis for computation of interactive networks within the Cytoscape software suite (National Institute of General Medical Sciences, Bethesda, MD, USA) [17] and design of an advanced visualization methodology. By exploiting this approach, we sought to significantly improve our ability to analyze such “-omics” data sets comprehensively and systematically and, in turn, to minimize the introduction of personal bias.

## Methods

### Animals

C57BL/6.NOD-*Aec1Aec2* and C57BL/6 male mice were bred and maintained under specific pathogen-free conditions at the Department of Pathology mouse facility at the University of Florida, Gainesville, FL, USA. To dissect the SGs, mice were killed by cervical dislocation after deep anesthetization. All procedures were approved by the University of Florida’s Institutional Animal Care and Use Committee (protocols B317-2007 and 2008011756).

### Isolation of RNA from salivary glands

Total RNA was isolated according to the protocol described in detail elsewhere [18]. When the mice were 4, 8, 12 and 16 weeks of age, the SGs free of lymph nodes were excised in parallel from five C57BL/6.NOD-*Aec1Aec2* and five C57BL/6 mice, then snap-frozen in liquid nitrogen. Total RNA from each mouse was isolated concurrently using the RNeasy Mini Kit (QIAGEN, Valencia, CA, USA), then RNA concentrations and purities were evaluated using UV spectroscopy. The ratio of absorbance (260 nm and 280 nm) of the RNA samples averaged 1.976. Subsequently, each sample was hybridized separately on a GeneChip Mouse Genome 430 2.0 Array and 3′ IVT Express Kit (Affymetrix, Santa Clara, CA, USA) according to the manufacturer’s instructions (annotation: build 32; 6 September 2011). Microarrays were assessed using Affymetrix Expression Console Software 1.1 without changing the default settings (Affymetrix), and the data quality was deemed adequate for further analyses.

### Submission of data to Gene Expression Omnibus

All the data sets reported herein have been deposited and are publicly available in the Gene Expression Omnibus [GSE15640, GSE36378].

### Verification of microarray data

In addition to the experiments performed to validate the quality of the microarray data presented previously [19,20], verification experiments were expanded to include groups of genes in accordance with the specific aims of this study. Real-time polymerase chain reactions (PCRs) were carried out using the Extracellular Matrix & Adhesion Molecules PCR Array (PAMM-013Z; SABiosciences, Valencia, CA, USA) and the PI3K-AKT Signaling PCR Array (PARN-058Z; SABiosciences) according to the instructions provided by the manufacturer. These arrays were analyzed using RT^2^ Profiler PCR Array Data Analysis software (SABiosciences) to calculate the fold changes in gene expression occurring within the respective time periods. These data were subsequently plotted against the values yielded by the GeneChip Mouse Genome 430 2.0 Array and 3′ IVT Express Kit array (Additional file 1: Figures S1A to S1C) and subjected to correlation analyses (Additional file 1: Figure S1D).

### Data analysis pipeline

**Figure 1:**
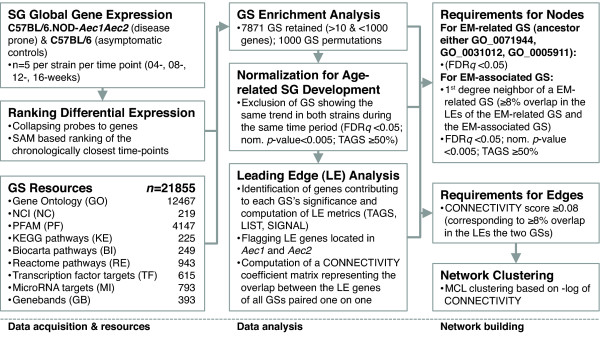
**Schematic representation of the data analysis and data visualization pipeline.** Designed for unbiased mapping of alterations in the transcriptional landscape of the extracellular milieu (EM). CONNECTIVITY, degree of overlap in LE-members between the GSs the edge is connecting; FDR, false discovery rate; GS, gene set; KEGG, Kyoto Encyclopedia of Genes and Genomes; LE, leading edge; LIST, percentage of genes in the ranked gene list before (for positive enrichment score (ES)) or after (for negative ES) the peak in the running ES (indication of where in the list the enrichment score is attained); MCL, Markov cluster algorithm; NCI, National Cancer Institute database; PFAM, Pfam protein families database [21]; SAM, significance analysis of microarrays; SG, salivary gland; SIGNAL, ES strength that combines LIST and TAGS; TAGS, percentage of gene hits before (for positive ES) or after (for negative ES) the peak in the running ES, (indicates percentage of genes contributing to ES).

A flow diagram of the data analysis pipeline is depicted in Figure 1.

### Normalization of microarray probe cell intensity files

Probe cell intensity files (.CEL) were quantile-normalized and underwent general background correction. Control metrics were generated and passed for each array (Robust Multichip Analysis performed with Affymetrix Expression Console Software 1.1; Affymetrix). Genes covered by multiple probes on the microarray chip were collapsed to genes by selecting the probe yielding the highest signal (J-Express 2009 software; MolMine AS, Hafrsfjord, Norway).

### Ranking differential gene expression between two biological states

To identify coordinated and significant changes between the two chronologically closest time points from each strain, the collapsed gene lists (21,673 genes) were ranked based on the observed relative difference upon performing significance analysis of microarrays (SAM) in J-Express 2009. SAM makes no assumption about the distribution of the data and effectively introduces a nonarbitrary fold increase criterion, thus superseding the introduction of a subjective fold-change threshold. These ranked lists were loaded into the GSEA v2.07 database (Broad Institute, Cambridge, MA, USA).

### Compilation of gene sets for gene set enrichment analysis

A gene set (GS) is an *a priori*-defined groups of genes compiled, curated and annotated to reflect one specific trait that its members share, such as they are all collagens [15]. The first GSs compiled for this study were extracted from the following bioinformatics resources as described previously [22]: (1) Gene Ontology (GO) (*n* = 12,467), (2) National Cancer Institute (NC) (*n* = 219), (3) PFAM (PF) (*n* = 4,147) and (4) Kyoto Encyclopedia of Genes and Genomes (KE) (*n* = 225). Using the WhichGenes 1.0 GS building tool [23], we compiled the remaining GSs from (5) BioCarta pathways (BC) (*n* = 249), (6) Reactome pathways (RE) (*n* = 943), (7) transcription factor (TF) binding motifs (mouse orthologs were inferred from human genes) (*n* = 615), (8) microRNA binding motifs as defined in the miRDB database (http://mirdb.org/miRDB/) (MI) (*n* = 793) and (9) close genomic localization (Ensembl genes in bands resource) (GB) (*n* = 393). All GSs (*N* = 21,855) were downloaded between 14 and 20 August 2011.

### Running gene set enrichment analysis and identification of leading edge genes

GSEA aids in overcoming the analytical challenges posed by pleiotropy, as genes are assigned to GSs that represent each of their traits, and by the fact that biological processes commonly depend on a coordinated change in the expression of several genes [15]. Statistical analyses are performed for each GS by assessing the expression pattern formed by its members within the entire data set (21,673 genes). Thus, an asymmetrical distribution skewed significantly to the overexpressed end of the ranked list signifies significant enrichment. In contrast, such an asymmetrical distribution indicates significant depletion in cases where the expression pattern of the GS is skewed significantly to the underexpressed end of the ranked list. This step of computational interpretation based on *a priori*-defined and consensus-based biological knowledge without setting arbitrary cutoffs, such as fold change or significance level, prevents the introduction of bias and increases the robustness and comparability of results. GSEA was performed for GSs larger than 10 and smaller than 1,000 (7,871 of 21,855 GSs retained). Permutation number was deemed adequate at 1,000 iterations, and default values were used for all other parameters.

LE analysis identifies the genes of each GS that appear in the ranked list at or before the point at which the running sum reaches its maximum deviation from zero. Hence, genes assigned to a GS’s LE (LE genes) are the genes accounting for the individual GS significant enrichment or depletion signal [15]. LE analyses were computed after GSEA using GSEA v2.07.

### Subtraction of alterations associated with age-related salivary gland development

**Figure 2:**
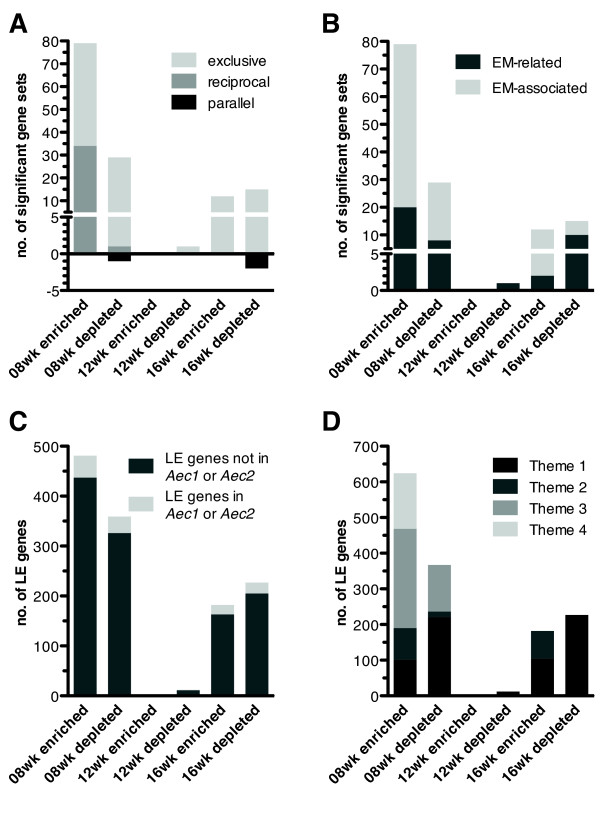
**Extent of significant alterations during emergence and onset of overt Sjögren’s syndrome. (A)** Number of exclusive GSs (GSs yielding significance in C57BL/6.NOD-Aec1Aec2 mice only), reciprocal GSs (GSs yielding significance in C57BL/6.NOD-Aec1Aec2 and C57BL/6 mice, but with opposite trends) and parallel GSs (GSs yielding significance in C57BL/6.NOD-Aec1Aec2 mice and C57BL/6 mice with the same trend (excluded)). **(B)** Number of extracellular milieu(EM)-related and EM-associated gene sets (GSs) significantly altered for each time period. **(C)** Number of leading edge (LE) genes underlying the changes displayed in **(A)**, categorized with respect to localization inside or outside the susceptibility regions *Aec1* and *Aec2*. **(D)** Number of LE genes underlying each of the major biological themes.

To normalize for changes in gene expression associated with normal SG activities, we discarded GSs that yielded significant enrichment or depletion in both strains in parallel and over the same period of time (false discovery rate (FDR) <0.05, nominal *P*-value <0.005, TAGS ≥ 50% in C57BL/6 mice) from all subsequent analyses (Figure 2A; parallel). Reciprocal changes over the same period of time (for example, enriched in C57BL/6.NOD-*Aec1Aec2* while depleted in C57BL/6 mice) were retained and, together with GSs uniquely altered in C57BL/6.NOD-*Aec1Aec2* mice (Figure 2A; exclusive), selected for network building.

### Network building

Network analysis is the study of a system that is depicted as connections (that is, edges) between discrete objects (that is, nodes). To define the EM, GSs that yielded a FDR less than 0.05 and had either GO_0071944 (cell periphery), GO_0031012 (ECM) or GO_0005911 (cell–cell junction) as an ancestor in the GO tree were selected and qualified as EM-related GSs.

GSs connected by an edge (≥8% of shared LE members) to an EM-related GS were qualified as an EM-associated process when they passed the significance criteria (FDR <0.05, nominal *P*-value <0.005, TAGS ≥50%). Defining the forces of attraction, the degree of overlap in LE members between the GSs also determined their position in the network computed using the edge-weighted, spring force–directed layout in Cytoscape 2.8.2 [17]. Cytoscape is an open source software platform utilized for visualizing complex networks and integrating these networks with any type of attribute data [17]. The connectivity parameter, defined by the degree of overlap in LE members between the GSs, could thereby also be applied as an edge weight for the subsequent MCL clustering [16] computed within Cytoscape. The clusters identified by the MCL are defined by simulating the stochastic flow within the networks [16].

## Results

### Extent of alterations across the three time periods

Application of the data analysis pipeline outlined in Figure 1 revealed that the most thematically diverse alterations specific for C57BL/6.NOD-*Aec1Aec2* mice, involving the most EM-related GSs and EM-associated GSs (Figure 2B), occurred between 4 and 8 weeks of age. The same was true for the number of genes accounting for the GSs’ significant enrichment or depletion, that is, LE genes (Figures 2C and 2D).

Significant enrichment at 8 weeks of age involved 79 GSs that depended on coordinated upregulation of 481 LE genes. Interestingly, 43% of these GSs were simultaneously becoming depleted in age-matched C57BL/6 mice (Figure 2A; reciprocal). Over the same period of time, downregulation of 359 LE genes led to significant depletion of 29 GSs (Figures 2B and 2C). Between 8 and 12 weeks of age, a single GS was becoming depleted (Figures 2A and 2B) in conjunction with downregulation of 12 LE genes (Figures 2C and 2D). The transition from 12 to 16 weeks of age, which chronologically coincided with the onset of overt SS-like disease in C57BL/6.NOD-*Aec1Aec2* mice, was marked by enrichment of 12 GSs comprising a total of 182 LE genes, as well as depletion of 15 GSs as a consequence of downregulation of 227 LE genes (Figures 2B and 2C).

### Major biological themes involving the extracellular milieu during emergence of Sjögren’s syndrome

#### Progression from pre- to subclinical disease occurs between 4 and 8 weeks of age

**Figure 3:**
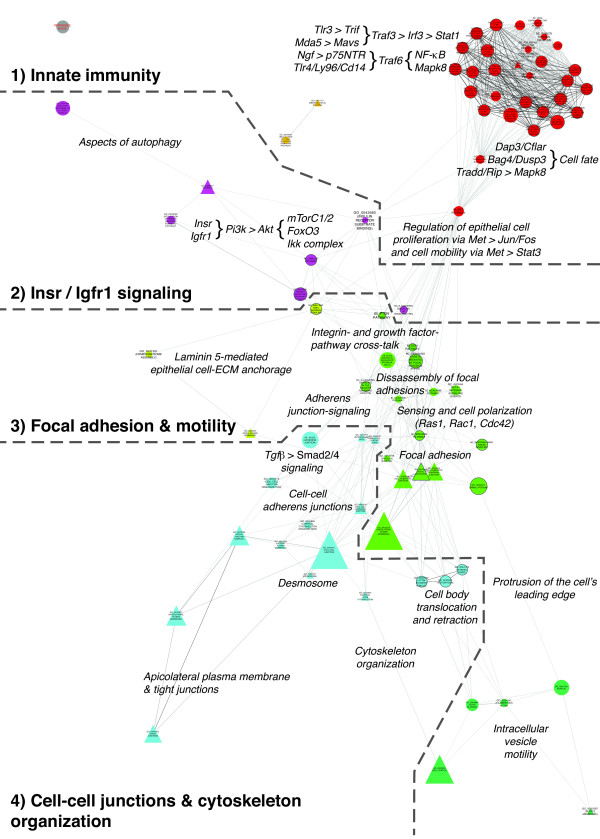
**Enrichments in the transcriptional landscape of the extracellular milieu during transition from pre- to subclinical Sjögren’s syndrome.** Gene sets (GSs) enriched at 8 weeks of age delineate activation of the innate immune system coinciding with significant alterations in the targeted tissue’s homeostasis and integrity. Proportions of leading edge (LE) genes shared between GSs defined distance, organization and clustering of the GSs. Dashed lines, separators between major biological themes; annotations in italics, interpretation of transcriptional activity inferred from the LE gene clouds displayed in Figure 4; node color, Markov cluster algorithm (MCL) cluster number. Node shapes: triangles, extracellular milieu (EM)–related; circles, EM-associated; node size, relative to number of detected genes that are members of this GS (reference node = 50 genes). Node label type size, relative to percentage of genes belonging to this GS’s LE (TAGS) (reference node = 75%). Node border: none, alteration of this GS exclusive to C57BL/6.NOD-*Aec1Aec2* mice; present, reciprocal trend in C57BL/6 mice. Edge color: degree of overlap in LE genes between the two GSs connected by this edge. ECM, extracellular matrix; Igfr1, insulin-like growth factor receptor 1; Insr, insulin receptor.

**Figure 4:**
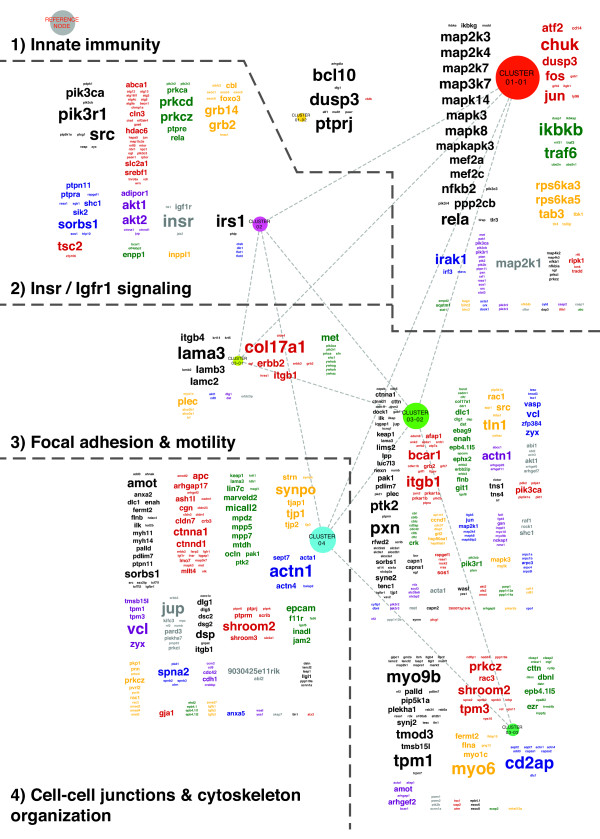
**Annotation of the Markov cluster algorithm clusters with their respective leading edge gene clouds.** The individual gene sets (GSs) of each Markov cluster algorithm cluster shown in Figure 3 were collapsed into a metanode. Network: metanode color represents the original color of the ancestor GSs and node size and node label font size are proportional to the number of GSs collapsed into this metanode (reference node = 15 GSs). Clustered leading edge (LE) gene clouds: Font color represents clustering of the LE genes based on the connections between the original GSs. Font size is proportional to the frequency of the gene in the LEs of the GSs collapsed into this metanode.

The network displaying all GSs enriched by 8 weeks of age (Figure 3), together with interpretation of the respective LE genes (Figure 4), allowed us to identify four major biological themes: (1) activation of pathways characteristic of innate immune responses to long double-stranded RNA viruses; (2) insulin receptor (Insr) and insulin-like growth factor 1 (Igfr1)-mediated signaling via phosphoinositide 3-kinase (PI3K) and protein kinase B (AKT) further guiding cell fate, proliferation and differentiation; (3) remodeling of epithelial cell–ECM anchorage via focal adhesions (FAs) whose specificities allowed bidirectional integrin growth factor signaling pathway cross-talk as well as all effector processes related to cell motility; and (4) the three major classes of intercellular junction complexes engaging in cell-cell signaling via E-cadherin (CDH1) and involving transforming growth factor β (TGFβ).

**Figure 5:**
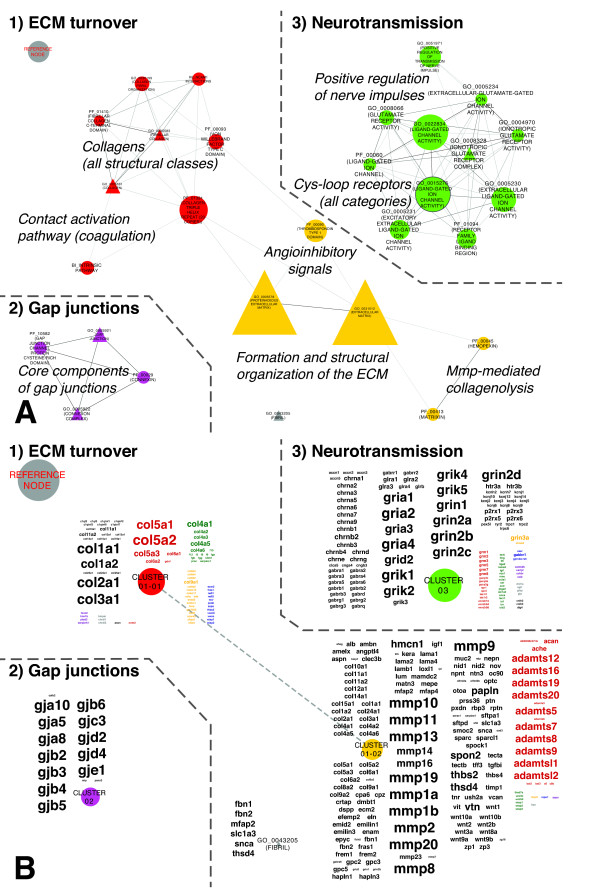
**Depletions in the transcriptional landscape of the extracellular milieu during transition from pre- to subclinical Sjögren’s syndrome. (A)** Gene sets (GSs) depleted when the mice were 8 weeks of age define marked deceleration of extracellular matrix (ECM) turnover and significantly decreased transcription of genes associated with gap junction formation and neurotransmission. The layout parameters of Figure 4A correspond precisely to the layout parameters of Figure 3. The reference node allows estimation of scaling and direct comparison of Figures 3, 5A, 6A and 7A and Additional file 1: Figure S2A. Mmp, matrix metalloproteinase. **(B)** Annotation of the Markov cluster algorithm clusters displayed in **(A)** with their respective leading edge gene clouds. The layout parameters correspond precisely to the layout parameters of Figure 4. The reference node allows estimation of scaling and direct comparison of Figures 4, 5B, 6B and 7B and Additional file 1: Figure S2B.

GSs that were depleted in 8-week-old C57BL/6.NOD-*Aec1Aec2* mice clustered in three independent networks (Figure 5A), each depending on distinct sets of LE genes (Figure 5B): (1) deceleration of ECM turnover, (2) downregulation of genes encoding gap junction proteins and (3) loss of positive regulation of nerve impulses in conjunction with downregulation of genes encoding members of all classes of cysteine (Cys) loop neurotransmitter receptors and fewer metabotropic receptors.

#### Stabilization of subclinical disease state between 8 and 12 weeks of age

GO_0005581 (COLLAGEN) was the only GS yielding significance during this time period (Additional file 1: Figure S2A) and representing 12 LE genes (Additional file 1: Figure S2B).

#### Transition from subclinical to overt disease between 12 and 16 weeks of age

**Figure 6:**
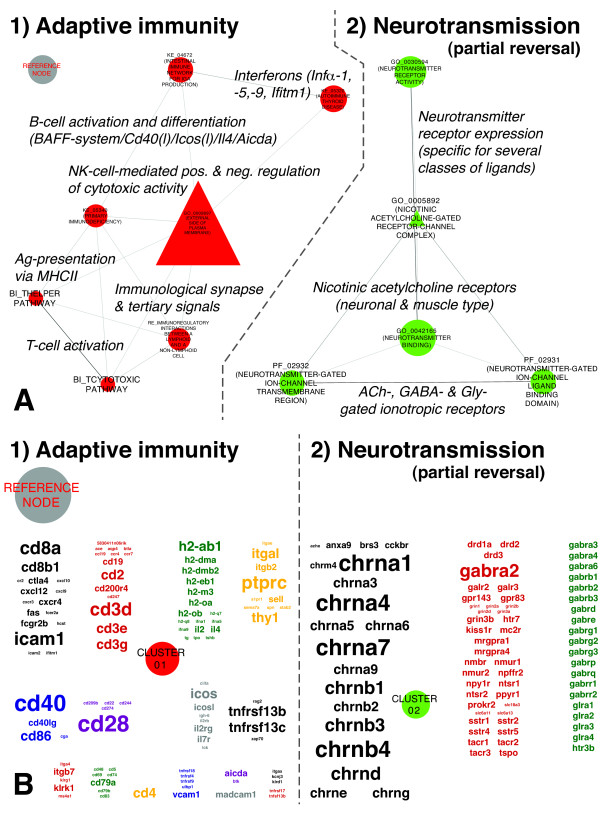
**Enrichments in the transcriptional landscape of the extracellular milieu during transition from subclinical to overt Sjögren’s syndrome. (A)** Gene sets (GSs) enriched at 16 weeks of age mirror the establishment of a pathogenic immune reaction in the targeted tissues and, in addition, reflect partial normalization of prior neurotransmitter receptor gene-associated alterations. The layout parameters correspond precisely to the layout parameters of Figure 3. The reference node allows estimation of scaling and direct comparison of Figures 3, 5A, 6A and 7A and Additional file 1: Figure S2A. ACh, acetylcholine; GABA, γ-aminobutyric acid; Gly, glycine; MHCII, major histocompatibility complex class II; NK, natural killer. **(B)** Annotation of the Markov cluster algorithm clusters displayed in Figure 5A with their respective leading edge gene clouds. The layout parameters of Figure 5B correspond precisely to the layout parameters of Figure 4. The reference node allows estimation of scaling and direct comparison of Figures 4, 5B, 6B and 7B and Additional file 1: Figure S2B.

The GSs enriched at 16 weeks of age (Figure 6A), in conjunction with their respective LE genes (Figure 6B), delineated two distinct themes: (1) emergence of an effector immune response characterized by reinforcement of the IFNα signature and a natural killer (NK) cell population, together with formation of the primary immunological synapse and late costimulatory signals delivering survival, proliferation and maturation signals to T cells and B cells; and (2) resumption of gene transcription for Cys loop receptors with acetylcholine (ACh), γ-aminobutyric acid (GABA) and glycine (Gly) binding specificities and initial upregulation of specific subsets of metabotropic receptors.

**Figure 7:**
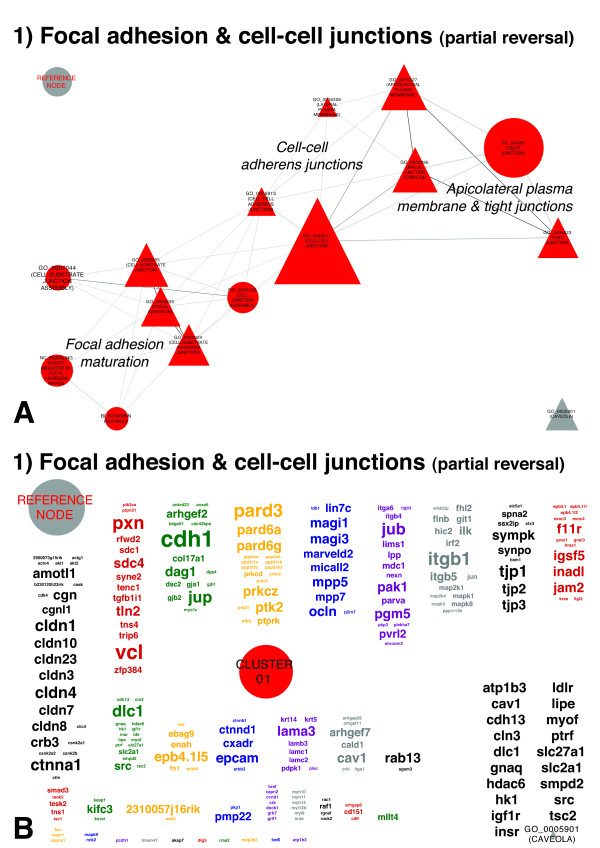
**Depletions in the transcriptional landscape of the extracellular milieu during transition from subclinical to overt Sjögren’s syndrome. (A)** Gene sets (GSs) depleted at 16 weeks of age signify, to a major extent, the partial reversal of enrichments in FAs and cell–cell junction-associated GSs observed at 8 weeks of age. The layout parameters of Figure 6A correspond precisely to the layout parameters of Figure 3. The reference node allows estimation of scaling and direct comparison of Figures 3, 5A, 6A and 7A and Additional file 1: Figure S2A. **(B)** Annotation of the Markov cluster algorithm clusters displayed in Figure 6A with their respective LE gene clouds. The layout parameters of Figure 6B correspond precisely to the layout parameters of Figure 4. The reference node allows estimation of scaling and direct comparison of Figures 4, 5B, 6B and 7B and Additional file 1: Figure S2B.

At 16 weeks of age, 80% of the depleted GSs (Figure 7A) and a large number of LE genes (Figure 7B) showed partial reversal of the alterations pertaining to FAs and cell–cell junctions observed earlier between 4 and 8 weeks of age (Figures 3 and 4). The remaining 20% of the GSs, such as those GSs not subject to earlier alterations, reinforced the cellular component (CC) terms GO_0005923 (TIGHT JUNCTION) and GO_0005925 (FOCAL ADHESION).

### Major biological themes dependent to significantly different degrees on genes located in *Aec1* and *Aec2*

**Figure 8:**
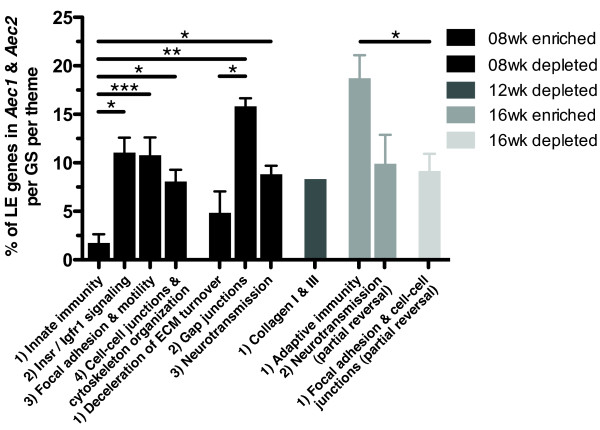
**Degree of dependence of the major biological themes on genes located in *Aec1 *and *Aec2 *(mean ± SEM).** Proportion of leading edge (LE) genes located in the disease-causing congenic loci *Aec1* and *Aec2*/gene set (GS)/major biological theme within the same disease phase were compared using one-way analysis of variance and Tukey’s *post hoc* test. *P*-values <0.05 were considered significant (*P < 0.05, **P < 0.01, ***P < 0.0001). Columns represent the means, and error bars represent the standard errors of the mean. ECM, extracellular matrix; Igfr1, insulin-like growth factor receptor 1; Insr, insulin receptor.

The quantitative contributions of the SS-predisposing genomic regions *Aec1* (chromosome 3; 0 to 46 cM) and *Aec2* (chromosome 1; 29.7 to 106.1 cM) to each GS are, together with the LE genes located in these susceptibility regions per biological theme, presented in Additional file 1: Figures S3 to S7. Comparing average proportions of LE genes located in *Aec1* and *Aec2* per GS and per biological theme showed that the innate immunity theme was least dependent (mean = 1.75%), and that the adaptive immunity theme was most dependent (mean = 18.71%), on LE genes located in *Aec1* or *Aec2* (Figure 8 and Additional file 1: Figures S3 and S6). Regarding the subclinical phase of SS, the greater reliance on genes located in the congenic regions of the themes associated with the SGs’ homeostasis and integrity compared to innate immunity may indicate that the latter occur subsequently and in response to these tissue-specific alterations (Figure 8 and Additional file 1: Figures S3 and S4).

### Detailed annotation of networks based on interpretation of gene set parameters and leading edge gene patterns

As described in the Methods section, the number of LE genes shared between GSs determined their position, interconnectivity and cluster membership in correspondence with all other GSs of a network. Thus, GSs in close proximity to each other share distinct similarities in their LE gene patterns. To resolve redundancies, commonly caused by GSs representing complex pathways, highly interconnected network areas require additional interpretation. The same accounts for large GSs annotated with terms too general to reflect the true theme shared by their LE members.

The basis for this curated annotation, written in italic type in Figures 3, 5A, 6A and 7A, is formulated upon analysis of (1) the LE members of each MCL cluster displayed as LE gene clouds generated by using a vector graphics–capable adaptation of the WordCloud Cytoscape plugin [24] (Figures 4, 5B, 6B and 7B), (2) each GS’s LE genes (Additional file 2) and (3) current literature–based interactome maps (Additional file 1: Figure S8). Additional file 3 comprises the networks displayed in Figures 3-7 as infinitely scalable and electronically searchable vector graphics, thereby allowing the visualization of network detail.

#### Transcriptional changes underlying themes being enriched during progression from pre- to subclinical Sjögren’s syndrome–like disease

In Figure 4, the LE gene cloud for Cluster_01-01, in combination with the percentage of each pathway covered by its LE members (Additional file 2; TAGS), points toward two pattern recognition receptors, namely, Toll-like receptor 3 (TLR3) and IFN-induced helicase C domain–containing protein 1 (IFIH1), also known as MDA5. Both these receptors are key molecules upstream of IFN regulatory factor 3 (Irf3) and signal transducer and activator of transcription 1 (STAT1) (Figure 4, Cluster_01-01). Also delineated by this cluster are upregulation of *Tlr4* and its coreceptors *Cd14* and lymphocyte antigen 96 (*Ly96*). These may, via their upregulated signaling cascade, deliver the strongest trigger for the observed canonical activation of nuclear factor κ-light-chain-enhancer of activated B cells (NF-κB) and mitogen-activated protein kinase 8 (MAPK8) [4,25]. In addition, the gene nerve growth factor (*Ngf*), which encodes another important inducer of NF-κB, was upregulated, even though NGF’s crucial receptor p75 neurotrophin receptor (p75NTR) [26] was absent from the list of LE genes for RE_P75NTR SIGNALS VIA NF-KB; TAGS = 73% (Additional file 2).

Determining the effect of the interconnecting NC_TRAIL SIGNALING PATHWAY; TAGS = 63% and BI_MET PATHWAY; TAGS = 74% GSs is more difficult because of incomplete coverage of the different arms of the TNF-related apoptosis-inducing ligand (TRAIL) pathway [27] and the hepatocyte growth factor receptor (MET) pathway [28] by their respective LE genes (Additional file 2). However, their involvement in determining cell fate and proliferation is reflected by their central position in Figure 3.

The GSs and LE genes associated with Cluster_02 (Figures 3 and 4) suggest that INSR and IGFR1, via their shared downstream signaling cascade involving PI3K and AKT, upregulate mammalian target of rapamycin complex 1 (mTorC1) and mTorC2. The mTor system in turn is pivotal in determining cell fate [29]. Increased autophagy is inferred by the presence of PI3K pathway members and several autophagy-related protein (*Atg*) encoding genes (Figure 4, Cluster_02).

In close proximity, Cluster_03-01 and Cluster_03-02 delineate cell-matrix adhesion complexes that transmit regulatory signals and mechanical forces (Figures 3 and 4) [30,31]. Cluster_03-01, including GO_0031581 (HEMIDESMOSOME ASSEMBLY); TAGS = 73%, defines the hemidesmosome-mediated, laminin-5-dependent anchorage of epithelial cells’ intermediate filaments to the basal lamina of the ECM. Cluster_03-02 represents, in large part, signaling pathways that are activated by alterations in a cell’s immediate surroundings and are transmitted via actin cytoskeleton–anchored FAs, such as NC_SIGNALING EVENTS MEDIATED BY FOCAL ADHESION KINASE; TAGS = 68% [31]. Thus, 31 of 77 genes annotated in the CC GO_0005925 (FOCAL ADHESION) GS were located in its LE (TAGS = 40%).

Matching the integrin genes (that is, *Itgav*, *Itgb1*, *Itgb4* and *Itgb5*) with the dominant growth factor receptor genes (that is, *Met*, *Insr*, *Igfr1*, fibroblast growth factor receptor 1 (*Fgfr1*) and *Tgfbr1*) in the LE profiles displayed in Figure 4 suggests that integrin α_v_β_5_, upstream of the enriched integrin-linked kinase signaling-associated GSs, provides a basis for IGFR1-integrin cross-talk [32]. Similarly, α_v_β_5_ and α_v_β_1_ may allow for TGFBR1 signaling by collaborating with integrin pathways (Figure 3) [33]. *Tgfb1*, *Tgfb2* and *Tgfb3*, together with the TF Smad family member 2 (*Smad2*) and *Smad4* downstream of *Tgfbr1* and the negative feedback–associated *Smad7*, are all present in the LE of Cluster_04 (Figure 4). The presence of osteopontin (*Spp1*), another ligand of integrins α_v_β_1_ and α_v_β_5_ in the LE of Cluster_03-02 (Figure 4), indicates that FA maturation may also occur in relation to innate immune cells.

Supporting a critical role of FA remodeling during this transition from pre- to subclinical SS-like disease, calpain 1 (*Capn1*) and *Capn2* (Figure 4), which regulate the dynamics of FA assembly and disassembly, are at the center of the two calpain-specific GSs (Figure 3). In addition, all other effector phases of non-muscle-cell movement are represented by GSs and LE genes of Cluster_03-02, Cluster_03-03 and the intercalated section of Cluster_04, respectively (Figures 3 and 4) [30].

The fourth biological theme shares 14.3% of its LE genes with Cluster_03-02 described above. This is due to molecular similarities between CC GO_0030055 (CELL-SUBSTRATE JUNCTION); TAGS = 41% and CC GO_0005913 (CELL-CELL ADHERENS JUNCTION); TAGS = 46%. Multiple LE genes belonging to the claudin and the occludin gene families further indicate increased formation of tight junctions at CC GO_0016327 (APICOLATERAL PLASMA MEMBRANE); TAGS = 51% [30]. These two types of cell–cell junction complexes depend critically on CDH1 expressed by epithelial cells [30]. Correspondingly, NC_E-CADHERIN SIGNALING IN THE NASCENT ADHERENS JUNCTION; TAGS = 60% and CDH1 anchorage-related GS GO_0017166 (VINCULIN BINDING); TAGS = 60% were significantly enriched and are mapped at the center of Figure 3. Furthermore, enrichment of GS GO_0030057 (DESMOSOME); TAGS 40% [30] delineates a third class of intercellular junction complexes associated with genes that are upregulated approximately 8 weeks prior to the onset of SS-like disease in C57BL/6.NOD-*Aec1Aec2* mice.

#### Transcriptional changes underlying themes being depleted during progression from pre- to subclinical Sjögren’s syndrome–like disease

In Figure 5A, CC term GO_0031012 (EXTRACELLULAR MATRIX) is located at the center of the first major biological theme, becoming depleted during this time period (Figure 5A). It was the largest GS that yielded significance in this study, with 159 of its 320 members contributing to its significance (TAGS = 50%). The LE genes grouped in Cluster_01-01 delineate broad downregulation of genes encoding collagens of the ECM (Figure 5B) [34]. Contributed by GS BI_INTRINSIC PATHWAY; TAGS = 73% and suggesting endothelial cell activation, this cluster also includes coagulation factor–encoding genes.

The LE gene cloud of Cluster_01-02 in Figure 5B lists genes associated with all categories of specialized ECM proteins [30]. These include laminin (LAM) encoding subunits, such as *Lama4* and *Lamb1*; proteoglycans, such as versican (*Vcan*); and glycoproteins, such as fibrillin 1 (*Fbn1*) and *Fbn2*. Genes coding for all matrix metalloproteinases (MMPs) capable of degrading collagens, as well as distinct members of the disintegrins and metalloproteinases with thrombospondin motif (ADAMTS) family, also contributed to the significance of the GSs grouped in Cluster_01-02 (Figure 5A). ADAMTS peptidases catalyze procollagens (for example, *Adamts3*) and inhibit angiogenesis (for example, *Adamts5*, *Adamts8*, *Adamts9* and *Adamts20*) [35]. Genes annotated as inducers of wingless-type mouse mammary tumor virus (MMTV) integration site family members (*Wnt*) (for example, Norrie disease (*Ndp*)), several Wnt genes (for example, *Wnt1*) and all Wnt1-inducible signaling pathway proteins (*Wisp1*, *Wisp2* and *Wisp3*) [30] completed the LE of Cluster_01-02. These changes complement the marked and broad deceleration of ECM turnover as a potential consequence of the ongoing innate immune response and/or delayed conclusion of developmental processes in the SGs of C57BL/6.NOD-*Aec1Aec2* mice.

The second theme delineates downregulation of genes associated with GSs annotating gap junction core proteins (for example, PF_00029 (CONNEXIN); TAGS = 67%) (Figures 5A and 5B) and thus represents the only class of cell-cell junctions not enriched at 8 weeks of age.

The third theme is dominated by genes coding for ligand-gated ion channels essential for neurotransmission (Figure 5B, Cluster_03) [36]. The largest part of these genes encodes subunits of anionic Cys loop receptors (GABA_A_ 12/12, GABA_A_-ρ 2/3 and GlyR 5/5), cationic Cys loop receptor subunits (serotonin-gated 5-HT3A and 5-HT3B and nicotinic ACh receptor 14/16 subunits), 18 of 20 ionotropic glutamate receptor subunits and ATP-gated channels P2X purinoceptors P2X1, P2X3, P2X5 and P2X6, as well as subsets of voltage-gated and acid-sensing potassium channels (for example, amiloride-sensitive cation channels 1 to 3 (ACCN1 to ACCN3) and ACCN5). The remaining clusters of this gene cloud represent mainly metabotropic receptors involved in sensory perception, whereas the LE genes associated with GO_0051971 (POSITIVE REGULATION OF TRANSMISSION OF NERVE IMPULSE; TAGS = 62%) also include inflammatory mediators such as IFNγ, tumor necrosis factor (TNF) and interleukin 6 (IL-6), all of which are known to decrease the threshold for nerve impulse generation (Additional file 2) [37].

#### Transcriptional changes underlying stabilization of subclinical disease between 8 and 12 weeks of age

LE genes associated with the continued depletion of GO_0005581 (COLLAGEN); TAGS = 46% encode all peptide chains for collagen type I, the most abundant collagen of the ECM, and collagen type III (Additional file 1: Figure S2B and Additional file 2). Collagen type IV, which has coverage of 67%, is associated with basal membranes [34].

#### Transcriptional changes underlying themes being enriched during transition from subclinical to overt Sjögren’s syndrome–like disease

In Cluster_01 of Figure 6A, GO_0009897 (EXTERNAL SIDE OF PLASMA MEMBRANE); TAGS = 33% interconnects the EM-associated GSs that delineate the adaptive effector immune response. The LE pattern of integrins (Cluster_01; Figure 6B) suggests an increase of CDH1 adhesive integrin αEβ7-expressing intraepithelial T cells, whereas CD11c, encoded by *Itgax* and *Itgb2*, points toward antigen-presenting cells (APCs) of myeloid origin [38]. The latter represent the most probable source for the concomitant increase in transcription of various INFα-encoding genes (*Infα-1*, *Infα-5*, *Infα-9* and *Ifitm1)* in the SGs of C57BL/6.NOD-*Aec1Aec2* mice (Figures 6A and 6B).

The establishment of a NK cell population in the targeted tissues is supported by several distinct LE members (Cluster_01; Figure 6B). Cytotoxicity-triggering receptors NKG2-D type II integral membrane protein (*Klrk1*), *Cd244* and its ligand encoded by UL16-binding protein 1 (*Ulbp1*) represent three key components of NK cells’ effector pathway. In contrast, the NK cell receptor complex encoded by killer cell lectinlike receptor subfamily D member 1 (*Klrd1*) and G member 1 (*Klrg1*) exert a regulatory anticytotoxic effect [38,39]. The LE genes *Cd244* and *Cd48*, in conjunction with *Cd2* and intercellular adhesion molecule 2 (*Icam2*), may further suggest regulation of CD8^+^ T cells by NK cells. Expression of major histocompatibility complex (MHC) and MHC-related genes, however, were skewed toward upregulation of MHC class II (MHCII) and MHCII invariant chain (*Cd74*) expression (Figure 6B).

The chemokine receptor-ligand profile characterizes emigration of multiple APC and lymphocyte populations (*Cxcr4*:*Cxcl12*), as well as reinforced recruitment of T-helper type 1 (T_H_1) cells, NK cells and plasmacytoid dendritic cells (*Cxcl3*:*Cxcl9*/*Cxcl10* and *Ccr7*:*Ccl19*) (Figure 6B) [38]. Immune cell homing may also be facilitated by increased expression of LE genes that encode mucosal vascular addressin cell adhesion molecule 1 (*MAdCAM-1*) and lymphocyte function–associated antigen 1 (*Lfa-1*), encoded by *Itgal* and *Itgb2* and *Icam1*.

ICAM1 and LFA-1 ligation is also critical for Cd28-dependent T-cell activation [38]. The pattern of LE genes encoding costimulatory molecules assigns importance to both the activating Cd28-dependent pathway and the inhibitory cytotoxic T-lymphocyte antigen 4 (Ctla4)–dependent pathway (Figure 6B; Cluster_01). Regarding the T-cell-associated central component of the immunological synapse, T-cell receptor (TCR) accessory proteins (for example, *Cd3*), TCR coreceptors *Cd4* and *Cd8* and TCR-associated molecules (for example, *Cd45* (*Ptprc*)) are also covered by the LE gene cloud of Cluster_01. The concomitant upregulation of *Il2*, *Il2rb* and *Il2rg*, as well as the presence of *Cd69*, represent effects downstream of T-cell activation [38]. Regulating activation of T-cell effector lineages at this stage may thereby involve the two LE gene B7 family members B and T lymphocyte attenuator (*Btla*) and Tnf receptor superfamily 18 (*Tnfrsf18*) (Figure 6B).

With respect to late costimulatory signals, *Cd40*:*Cd40lg* and inducible T-cell costimulator (*Icos)*:*IcosL* are the receptor-ligand pairs present in the LE of Cluster_01 (Figure 6B). Both these systems, together with LE-gene *Il4*, are critical for mounting effective T_H_2 responses [38].

B-cell-specific genes (for example, immunoglobulin heavy constant μ (*Igh-6*), *Cd79a*, *Cd79b*, *Cd19* and *Cd22*) are highly represented in the LE gene cloud of Cluster_01 (Figure 6B). Increased transcription of *Tnfrsf13C* (that is, *Baffr*), *Tnfrsf17* (that is, *Bcma*) and *Tnfrsf13B* (that is, *Taci*), together with their common ligand *Tnfsf13b* (that is, *Baff*), as well as the activation-induced cytidine deaminase gene (*Aicda)* (Figure 6B and Additional file 2; LE gene list for KE_04672 (INTESTINAL IMMUNE NETWORK FOR IGA PRODUCTION); TAGS = 60%), further indicates strong signaling for survival, proliferation and differentiation of B cells in SGs marked by overt disease [38,40].

The second biological theme enriched by 16 weeks of age pertains to neurotransmission and marks a partial reversal of changes that occurred earlier in the disease course. Of the 78 LE genes defining enrichment at this later stage (Figure 6B; Cluster_02), 40 were previously associated with depletion of GSs concerning neurotransmission at 8 weeks of age (Figure 5B; Cluster_03). Reinitiating transcription are mainly ACh-, GABA- and Gly-gated ionotropic receptors coding genes (Additional file 2). Overlaps were also found for genes encoding receptors for dopamine (that is, *Drd1a*) and substance P (that is, *Tacr1*). Unique to enrichment at 16 weeks of age were genes encoding for metabotropic receptors specific for ACh (that is, *Chrm4*) and somatostatin (that is, *Sstr1*, *Sstr2*, *Sstr4*, *Sstr5*) (Figure 6B and Additional file 2) [36].

#### Transcriptional changes underlying themes being depleted during transition from subclinical to overt Sjögren’s syndrome–like disease

GSs (Figure 7A) and their LE genes (Figure 7B) depleted and downregulated, respectively, during this time period, predominantly signify the reversal of previous enrichments in FAs and cell-cell junction–associated GSs observed at 8 weeks of age (Figures 3 and 4). Pairwise comparison of the overlapping GSs revealed that, on average, 54% of LE genes contributing to depletion at 16 weeks of age also contributed to these GSs’ prior enrichment at 8 weeks of age. The highest percentage of LE members following this pattern was identified for NC_SIGNALING EVENTS MEDIATED BY FOCAL ADHESION KINASE, with 79%, and the lowest percentage was found for GO_0043296 (APICAL JUNCTION COMPLEX), with 43% (Additional file 2). The LE genes not included in these LE overlaps did not define additional biological themes, but instead contributed predominantly to the increased average coverage of the EM-related GSs at 16 weeks (TAGS 45%) compared to 8 weeks of age (TAGS 38%) (Additional file 2).

## Discussion

Although the technology for generating global gene expression profiles has matured, analysis and interpretation of these data sets still pose great challenges. This is also true with regard to delineating the underlying biological and chronological complex changes in biological states, such as covert stages of autoimmunity in an organ subsequently targeted by an autoimmune disease. Thus, the possibility of assessing all relationships among all components of a biological system simultaneously with an integrated and standardized concept such as the one presented herein meets a clear demand [41].

With respect to the immune system–specific findings of this study, considering the presence of IFN signatures in patients with SS [11], enrichment of innate immune response pathways at 8 weeks of age in C57BL/6.NOD-*Aec1Aec2* mice was anticipated. In addition, the molecular basis underlying the effector immune response at disease onset mimicked all major aspects of sialadenitis described in patients with SS [9]. Further validating our findings is that C57BL/6.NOD-*Aec1Aec2* mice, during their spontaneous and slow development toward overt disease, displayed alterations in biological pathways that, if knocked out or overexpressed from birth on a healthy genetic background, induce aspects of SS. These models of SS include mice deficient for NF-κB feedback regulation (C57BL/6.*IκBα*^*M/M*^) and mice transgenic for *Baff*[7].

Direct comparison of the results presented herein with the conclusions formulated subsequent to analyses using conventional “top gene list” approaches [19,20] defines the added value of this systems biology–based methodology as follows. (1) Focusing on the transcriptional landscape related to and associated with the EM seemed adequate to map, in its entirety and in a standardized fashion, the alterations in the SG’s decision-making processes associated with the emergence of autoimmunity in this model. Emphasizing the EM prevented the mapping of some of previously documented downstream effects induced via signals transmitted by the EM, however [19,20]. (2) The early activation of the innate immune system described herein represents a crucial feature which has not previously been reported in the context of this data set. (3) By applying this methodology, the data set could be interpreted in significantly more detail, which subsequently could be combined to present a more comprehensive picture.

**Figure 9:**
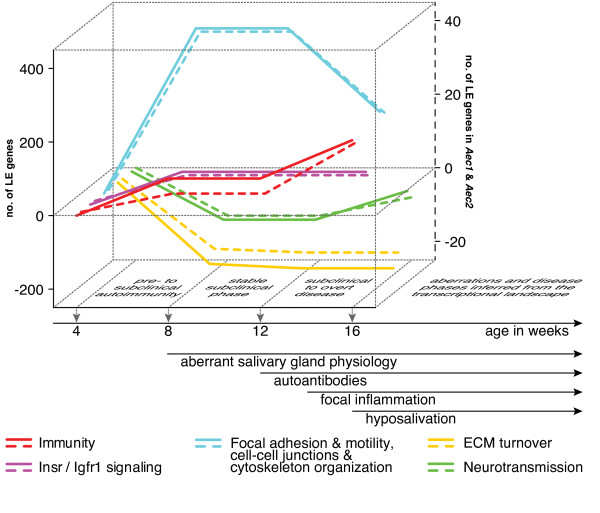
**Summary of the interrelationships between the major biological themes defining the alterations in the extracellular milieu during the emergence of Sjögren’s syndrome.** Time points are aligned in correspondence with the chronology of the development of major features of Sjögren’s syndrome in C57BL/6.NOD-*Aec1Aec2* mice [8]. Plotted on the *y*-axis is the cumulative number of leading edge (LE) genes (solid lines) associated with enrichment and depletion of the respective summarized biological theme over time. The right *y*-axis represents the same parameter, but for the LE genes specifically located in the disease-causing congenic loci *Aec1* and *Aec2* (dashed lines)*.* The summarized biological theme *Immunity* comprises the themes Innate immunity (Figure 3) and Adaptive immunity (Figure 6). The summarized biological theme *Focal adhesion and motility*, *cell–cell junctions and cytoskeleton organization* comprises the themes Focal adhesion and motility (Figure 3), Gap junctions (Figure 5) and Focal adhesion and cell-cell junctions (Figure 7). The summarized biological theme *Insr/Igfr1 signaling* comprises the theme Insr/Igfr1 signaling (Figure 3). The summarized biological theme *ECM turnover* comprises the themes ECM turnover (Figure 5) and Collagen I and III (Additional file 1: Figure S2). The summarized biological theme *Neurotransmission* comprises the themes Neurotransmission in Figures 5 and 6. ECM, extracellular matrix.

The transcriptional landscape of the EM of tissues targeted by autoimmunity described herein opens a novel and integrative perspective on the development of autoimmune diseases that might be of more general relevance (Figure 9) [42]. As a first step, it will be important to investigate how strongly, in other experimental models of autoimmunity, the LE genes differ. The chronological interrelationships and major biological themes identified herein may be the same, however. This knowledge may prove especially critical when aiming to delineate, on a systems level, the mechanisms of action and the targeted organ’s state subsequent to experimental immunomodulatory intervention [43].

The chronology of the etiopathology defined herein establishes several important points. First, long before overt experimental SS, susceptibility loci-dependent and partially transient alterations associated with the targeted tissue’s homeostasis and integrity formed the basis for an innate immune reaction. The latter, in contrast, was dependent predominantly on genes descending from the asymptomatic C57BL/6 strain that served as a genetic background for the generation of C57BL/6.NOD-*Aec1Aec2* mice. If a role of genes governing the SGs’ homeostatic state at such an early stage of autoimmunity can be confirmed, these genes may indeed crucially contribute to an individual’s risk of developing SS [5]. Second, the long-lasting, stable subclinical disease state may elucidate novel diagnostic strategies for identification of SS at an earlier state and thereby enable timely immunomodulatory treatment [44]. Third, major themes that defined this stable subclinical disease were abandoned concomitantly with the onset of overt disease. This permits speculation about whether these transient alterations may represent processes initiated by the SGs to resolve environmental challenges or to compensate for developmental deficiencies, primarily without involvement of the adaptive immune system. Fourth, LE gene patterns associated with costimulatory signals revealed both effector and regulatory ligand-receptor pairs’ being present, indicating that effector as well as immunoregulatory processes govern the onset of overt disease [45].

Although global data sets are seldom adequate to define the role of a single gene or protein, the isolated study of individual components in turn is limited in terms of elucidating how properties of biological systems emerge as a result of coordinated interactions between its numerous members and processes [41]. To take full advantage of the unbiased nature of “-omics” data sets, our concept integrates data analysis by relying extensively on bioinformatics resources for compilation of consensus-based, *a priori*-defined biological knowledge with an interactive model for data interpretation based on networks computed entirely from experimental data. Importantly, this concept is transferable to global data sets of any nature and achieves an important reduction in the number of arbitrary cutoffs set at the stage of data analysis. It also diminishes significantly the amount of personal bias commonly introduced during the process of data interpretation [46] and overcomes the confines of lists and matrices, which have clear limitations in conveying large amounts of complex data and interrelationships [47].

Obviously, to base such mappings on additional dimensions, such as global protein synthesis or posttranscriptional modification profiles, would significantly improve the validity of such analyses. They will become more feasible technologically and economically in the future [48]. In this study, we have computed a meaningful basis that has allowed us to formulate conclusions in agreement with the generality of our aim. In the future, assigning specific weights to the individual genes based on their uniqueness or importance to a specific GS may further standardize and facilitate the final steps of data interpretation. In the meantime, it is important to provide additional user-friendly graphical layouts of the networks, such as the ones presented herein, to enable the reader to scrutinize the authors’ detailed interpretation of the networks.

## Conclusions

By adhering to the principles of systems biology and adapting bioinformatics-based methodologies and data visualization to suit our aims, this study has delineated a novel perspective on the chronology and interplay between the SGs’ EM and the role of the innate and adaptive immune systems during the emergence of spontaneous, experimental SS (Figure 9). The timeline defined herein highlights the importance of genes governing the target tissue’s homeostatic state in establishing a stable subclinical disease state long before the clinical manifestation of SS. Formulating conclusions in agreement with the generality of our aim was possible only after having developed and applied the integrated data analysis and data visualization pipeline, which is also presented herein. This data-driven approach advances systematic and impartial interpretation of global datasets on the background of standardized, consensus-based, *a priori*-defined biological knowledge. It is widely applicable to the fields of immunology and rheumatology and will greatly facilitate analysis of complex alterations in biological states on a systems level, such as changes induced as a consequence of experimental treatment interventions.

## Abbreviations

ACh: Acetylcholine; ADAMTS: A disintegrin-like and metalloprotease with thrombospondin motifs; AICDA: Activation-induced cytidine deaminase gene; AKT: Protein kinase B; Atg: Autophagy-related protein; BTLA: B7 family member B- and T-lymphocyte attenuator; Capn: Calpain; CDH1: E-cadherin; Ctla4: Cytotoxic T-lymphocyte antigen 4; ES: Enrichment score; FBN1: Fibrillin; Fgfr1: Fibroblast growth factor receptor 1; GABA: γ-aminobutyric acid; Gly: Glycine; Icam: Intercellular adhesion molecule; Icos: Inducible T-cell costimulator; IFIH1: Interferon-induced helicase C domain–containing protein 1; IFN: Interferon; Igfr1: Insulin-like growth factor receptor 1; IgH-6: Immunoglobulin heavy chain, type μ; IL: Interleukin; Insr: Insulin receptor; IRF3: Interferon regulatory factor 3; ITG: Integrin; KLRD1: Killer cell lectinlike receptor subfamily D member 1; KLRG1: Killer cell lectinlike receptor subfamily G member 1; KLRK1: Cytotoxicity-triggering receptor; NKG2-D: Type II integral membrane protein; LAM: Laminin; LFA-1: Lymphocyte function–associated antigen 1; Ly96: Lymphocyte antigen 96; MadCAM1: Vascular addressin cell adhesion molecule 1; MAPK8: Mitogen-activated protein kinase 8; MET: Hepatocyte growth factor receptor; MMP: Matrix metalloproteinase; MMTV: Mouse mammary tumor virus integration site family member; mTorC: Mammalian target of rapamycin complex; NDP: Norrie disease; NF-κB: κ-light-chain-enhancer of activated B cells; Ngf: Nerve growth factor; PI3K: Phosphoinositide 3-kinase; P2X: ATP-gated channels P2X purinoceptor; p75NTR: p75 neurotrophin receptor; Smad: Smad family member; Spp1: Osteopontin; STAT: Signal transducer and activator of transcription; TAGS: Percentage of gene hits before (for positive enrichment score) or after (for negative enrichment score) peak in running enrichment score, which indicates percentage of genes contributing to enrichment score; Tgfβ: Transforming growth factor β; TLR: Toll-like receptor; TNF: Tumor necrosis factor; TNFRSF: Tumor necrosis factor receptor superfamily; TRAIL: Tumor necrosis factor–related apoptosis-inducing ligand; Ulbp1: UL16 binding protein 1; Vcan: Versican; Wisp: Wingless-type MMTV Integration site family 1-inducible-signaling pathway protein; Wnt: Wingless-type MMTV Integration site family.

## Competing interests

The authors have no financial or nonfinancial competing interests to declare.

## Authors’ contributions

ND conceived and implemented the data analysis and data visualization pipeline, analyzed and interpreted the data, created all the figures and wrote the manuscript. CQN took part in designing the study, carried out the animal experiments as well as the remaining experiments in the laboratory, and was involved in drafting the manuscript. KMT conceived and successfully adapted the WordCloud plug-in and was involved in drafting the manuscript. RJ contributed to the conception of the study, was involved in revising the manuscript and coordinated the project. ABP conceived the study, planned the animal experiments, participated in analyzing and interpreting the data and wrote the manuscript. All authors read and approved the final manuscript.

## Supplementary Material

Additional file 1 **Figure S1.** Verification of the microarray data. **Figure S2.** Stabilization of the subclinical disease state between 8 and 12 weeks of age. **Figures S3 to S7.** Contributions of genes located in susceptibility regions *Aec1* and *Aec2* to enrichments and depletions associated with transition from pre- to subclinical Sjögren’s syndrome (SS) and to onset of overt SS, respectively. **Figure S8.** Interactome model based on the leading edge members associated with enrichments in the transcriptional landscape of the extracellular milieu during transition from pre- to subclinical Sjögren’s syndrome between 4 and 8 weeks of age. Click here for file

Additional file 2 **All node parameters, LE genes and LE genes located in susceptibility regions *Aec1 *** **and *Aec2 *** **in tabular form.** Click here for file

Additional file 3 **The networks displayed in Figures **3**-**7** as infinitely scalable and electronically searchable vector graphics.** Click here for file
