# Supplementary material for: Transcriptional landscapes of emerging autoimmunity: transient aberrations in the targeted tissue’s extracellular milieu precede immune responses in Sjögren’s syndrome
Source: Arthritis Res Ther. 2013 Oct 31;15(5):R174. doi: 10.1186/ar4362 (PMC3978466; doi:10.1186/ar4362)
Supplement: Additional file 1 — Figure S1. Verification of the microarray data. Figure S2. Stabilization of the subclinical disease state between 8 and 12 weeks of age. Figures S3 to S7. Contributions of genes located in susceptibility regions Aec1 and Aec2 to enrichments and depletions associated with transition from pre- to subclinical Sjögren’s syndrome (SS) and to onset of overt SS, respectively. Figure S8. Interactome model based on the leading edge members associated with enrichments in the transcriptional landscape of the extracellular milieu during transition from pre- to subclinical Sjögren’s syndrome between 4 and 8 weeks of age. [file ar4362-S1.pdf]

Additional file 1

Figure S1)

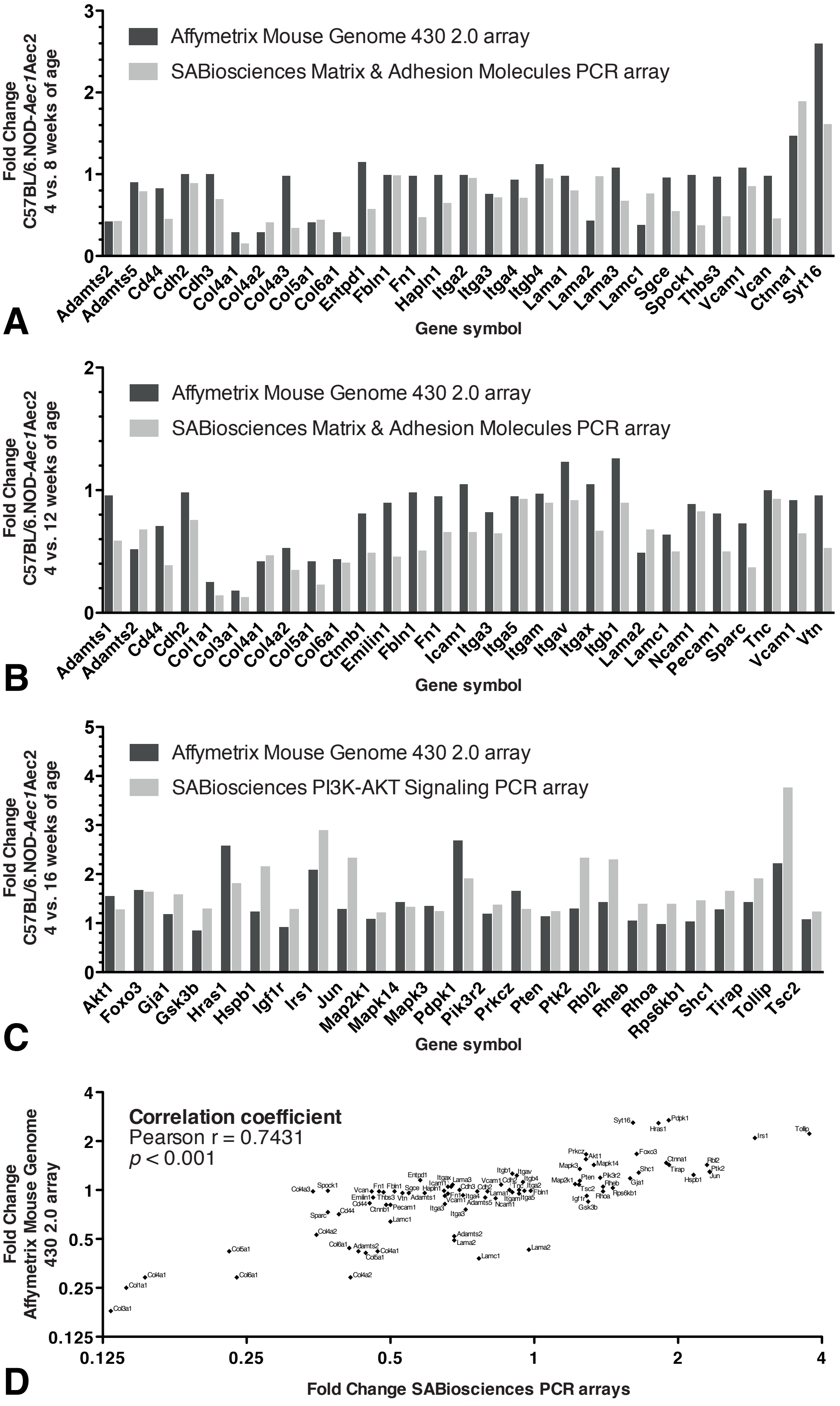

Comparing fold changes in gene expression over time inferred from microarray and real-time PCR data. A-C) Fold changes in gene expression as yielded by the SABiosciences Extracellular Matrix & Adhesion Molecules PCR array and SABiosciences PI3K-AKT Signaling PCR array, respectively compared to fold changes computed based on the Affymetrix 3' Expression Array GeneChip Mouse Genome 430 2.0 array data. **D)** Scatter plot of the data displayed in Supplementary Figures 1A-1C. Statistical analyses demonstrate a significant correlation between the fold changes obtained with the two methods (Pearson r = 0.7431; $p < 0.001$).

Figure S2)

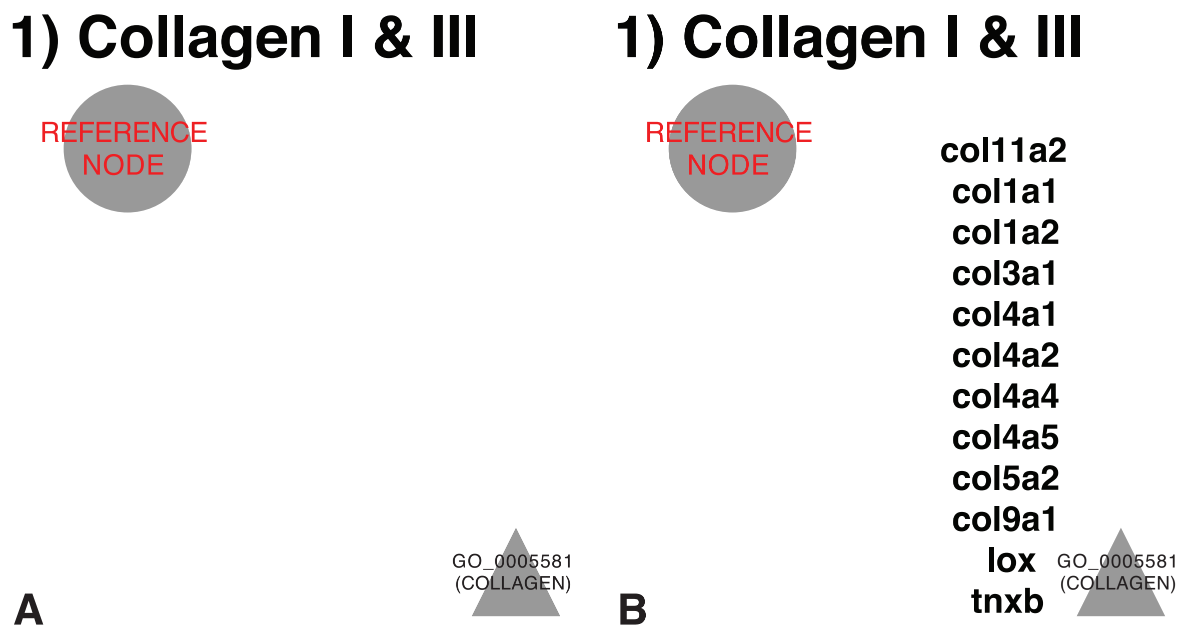

Stabilization of the subclinical disease state in C57BL/6.NOD-*Aec1Aec2* mice between 8 and 12 weeks of age. GO_0005581 (COLLAGEN) was the only GS, which yielded significance during this time period by continuing its depletion initiated between 4 and 8 weeks of age already. The LE-genes indicate collagen I & III to be important. **A)** The layout parameters of Figure S2A correspond precisely to the layout parameters of Figure 3. The reference node allows estimation of scaling and direct comparison of Figures 3, 5A, 6A, 7A & Figure S2A. **B)** Annotation of the GS displayed in Figure S2A with its respective LE-gene cloud. The layout parameters of Figure S2B correspond precisely to the layout parameters of Figure 4. The reference node allows estimation of scaling and direct comparison of Figures 4, 5B, 6B, 7B & Figure S2B. Figure infinitely scalable and electronically searchable.

Figure S3)

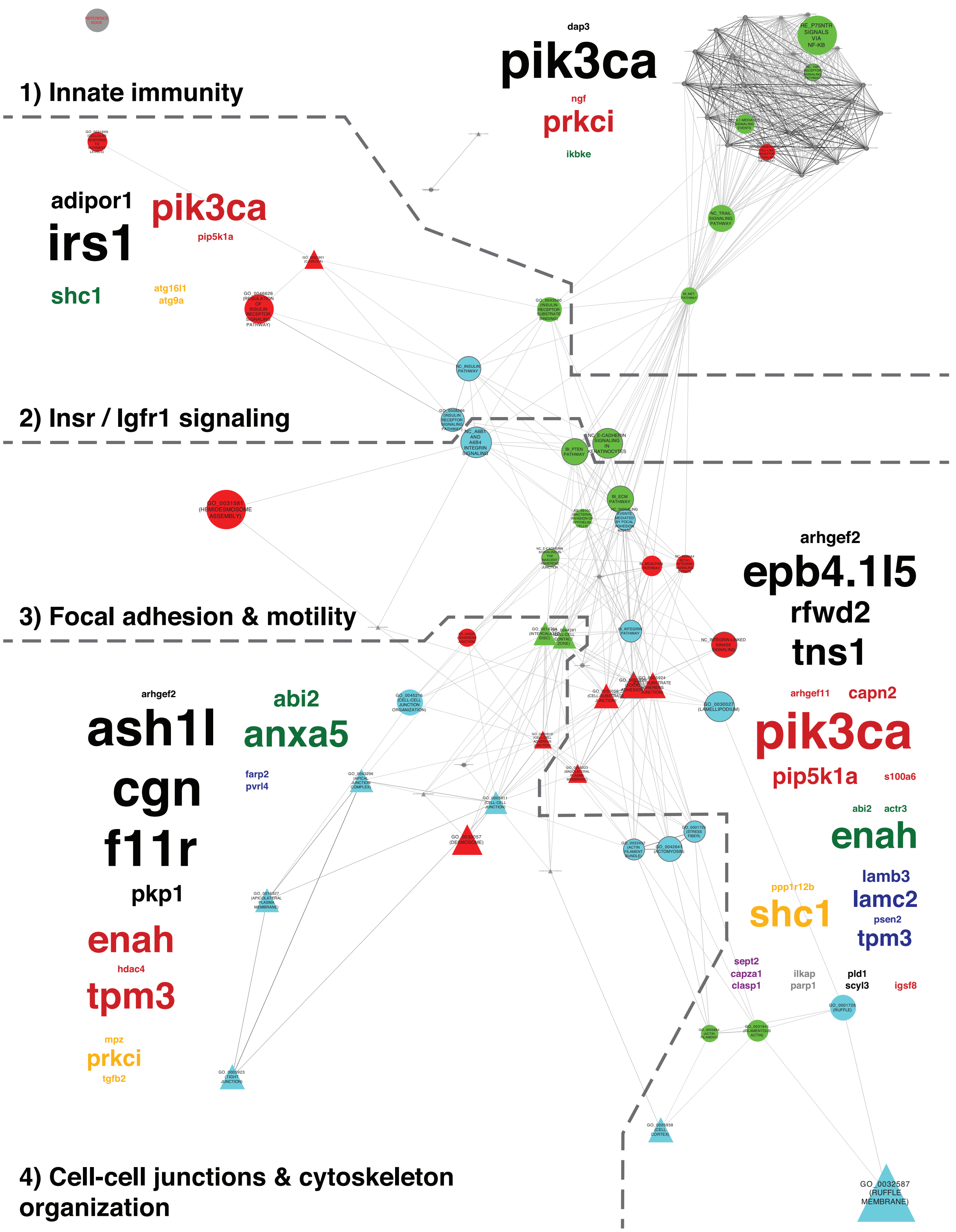
1) Innate immunity
dap3
pik3ca
ngf
prkci
ikbke
adipor1
irs1
pik3ca
pip5k1a
shc1
atg16l1
atg9a
2) Insr / Igfr1 signaling
3) Focal adhesion & motility
arhgef2
epb4.1l5
rfwd2
tns1
arhgef11
capn2
pik3ca
pip5k1a
s100a6
abi2
actr3
enah
ppp1r12b
shc1
lamb3
lamc2
psen2
tpm3
sept2
capza1
clasp1
ilkap
parp1
pld1
scyl3
igsf8
arhgef2
ash1l
cgn
f11r
pkp1
enah
hdac4
tpm3
mpz
prkci
tgfb2
abi2
anxa5
farp2
pvrl4
GO_0031581 (HEMIDESMOSOME ASSEMBLY)
GO_0032587 (RUFFLE MEMBRANE)
4) Cell-cell junctions & cytoskeleton organization

Contributions of genes located in susceptibility regions *Aec1* and *Aec2* to enrichments in the EM's transcriptional landscape during transition form pre- to subclinical SS in C57BL/6.NOD-*Aec1Aec2* mice between 4 and 8 weeks of age. Dashed line = separator between the major biological themes; **Node color:** green = LE-genes located in *Aec1*; red = LE-genes located in *Aec2*, blue = LE-genes located in both congenic regions, grey = no LE-genes located in *Aec1* or *Aec2*; **node size and node label font size** = proportional to the percentage of LE-genes located in the congenic regions (Reference Node = 10%); **node shape:** triangular = EM-related, circular = EM-associated; **node boarder:** none = alteration of this GS exclusive to C57BL/6.NOD-*Aec1Aec2* mice, present = reciprocal trend in C57BL/6 mice. **Edge color** = degree of overlap in LE-genes between the two GSs connected by this edge. **Clustered LE-gene clouds: Font color** = clustering of the LE-genes located in the susceptibility regions based on the connections between the GSs of the respective major biological theme; **font size** = frequency of this gene in the LEs of the GSs combined in the respective major biological theme. Figure infinitely scalable and electronically searchable.

Figure S4)

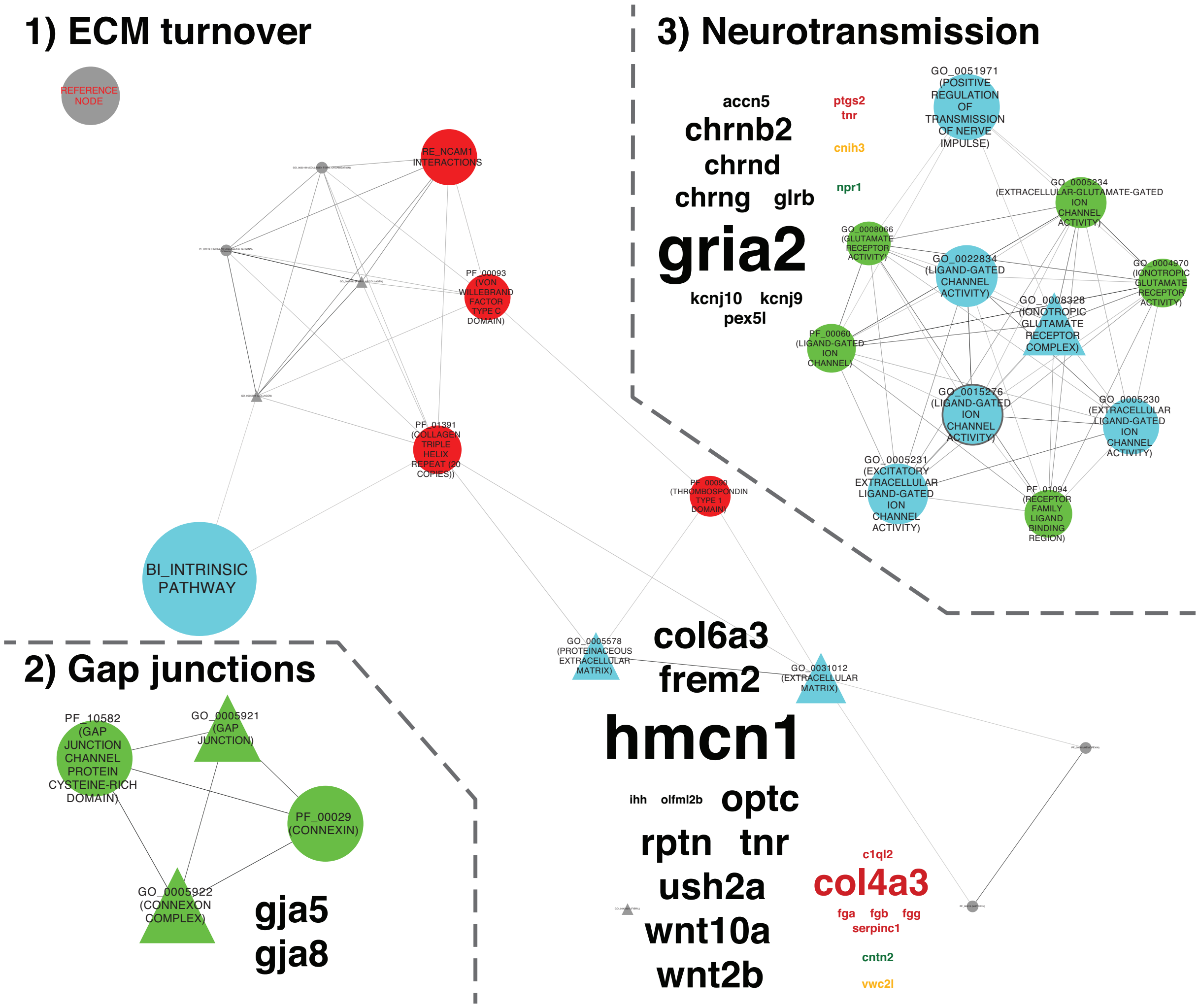

Contributions of genes located in susceptibility regions *Aec1* and *Aec2* to depletions in the EM's transcriptional landscape during transition form pre- to subclinical SS in C57BL/6.NOD-*Aec1Aec2* mice between 4 and 8 weeks of age. The layout parameters of Figure S4 correspond precisely to the layout parameters of Figure S3. The reference node allows estimation of scaling and direct comparison of Supplementary Figures 3-7. Figure infinitely scalable and electronically searchable.

Figure S5)

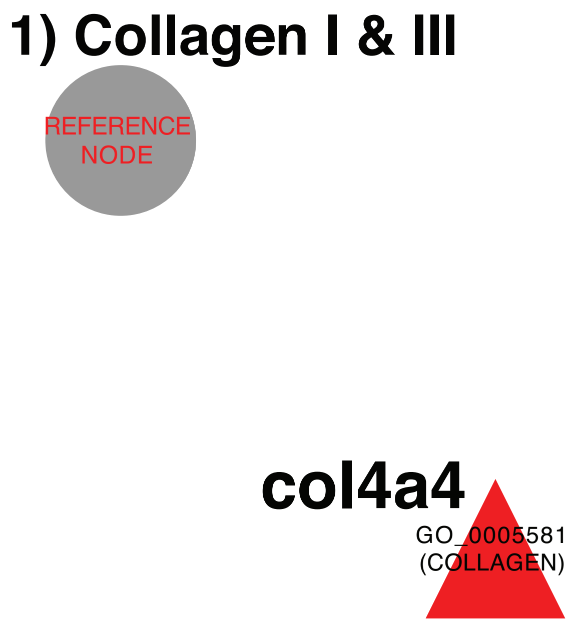

Contribution of genes located in the susceptibility regions *Aec1* and *Aec2* to the stabilization of the subclinical disease state in C57BL/6.NOD-*Aec1Aec2* mice between 8 and 12 weeks of age. The layout parameters of Figure S5 correspond precisely to the layout parameters of Figure S3. The reference node allows estimation of scaling and direct comparison of Supplementary Figures 3-7. Figure infinitely scalable and electronically searchable.

Figure S6)

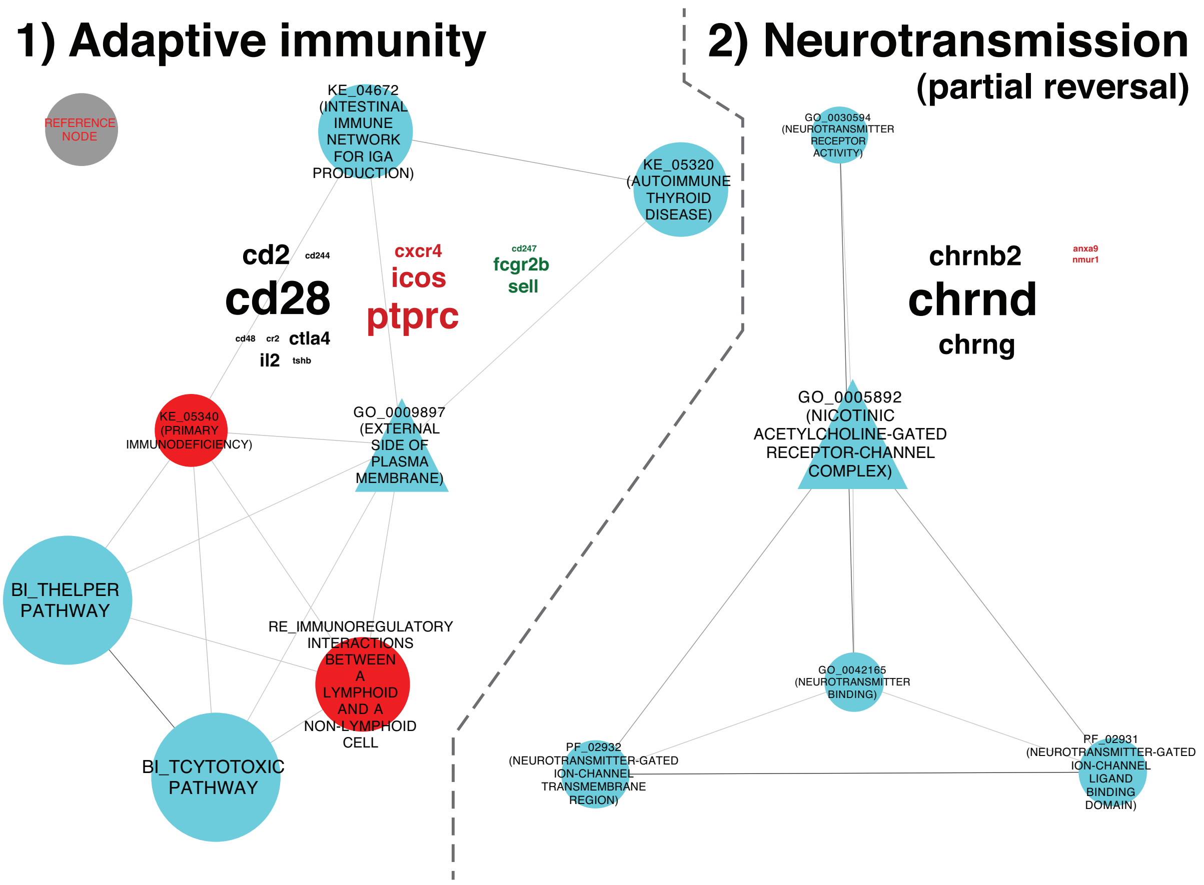

Contributions of genes located in susceptibility regions *Aec1* and *Aec2* to enrichments in the EM's transcriptional landscape during transition form subclinical to overt SS in C57BL/6.NOD-*Aec1Aec2* mice between 12 and 16 weeks of age. The layout parameters of Figure S6 correspond precisely to the layout parameters of Figure S3. The reference node allows estimation of scaling and direct comparison of Supplementary Figures 3-7. Figure infinitely scalable and electronically searchable.

Figure S7)

1) Focal adhesion & cell-cell junctions (partial reversal)

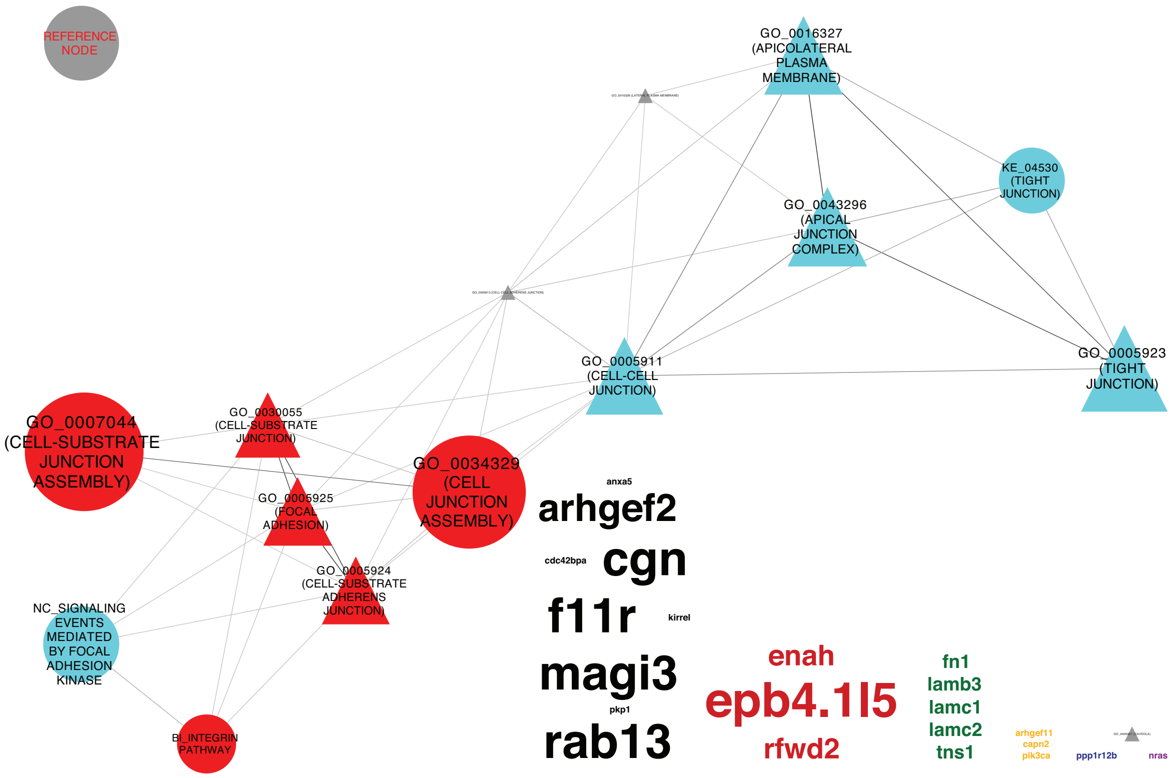

Contributions of genes located in susceptibility regions *Aec1* and *Aec2* to depletions in the EM's transcriptional landscape during transition form subclinical to overt SS in C57BL/6.NOD-*Aec1Aec2* mice between 12 and 16 weeks of age. The layout parameters of Figure S7 correspond precisely to the layout parameters of Figure S3. The reference node allows estimation of scaling and direct comparison of Supplementary Figures 3-7. Figure infinitely scalable and electronically searchable.

Figure S8)

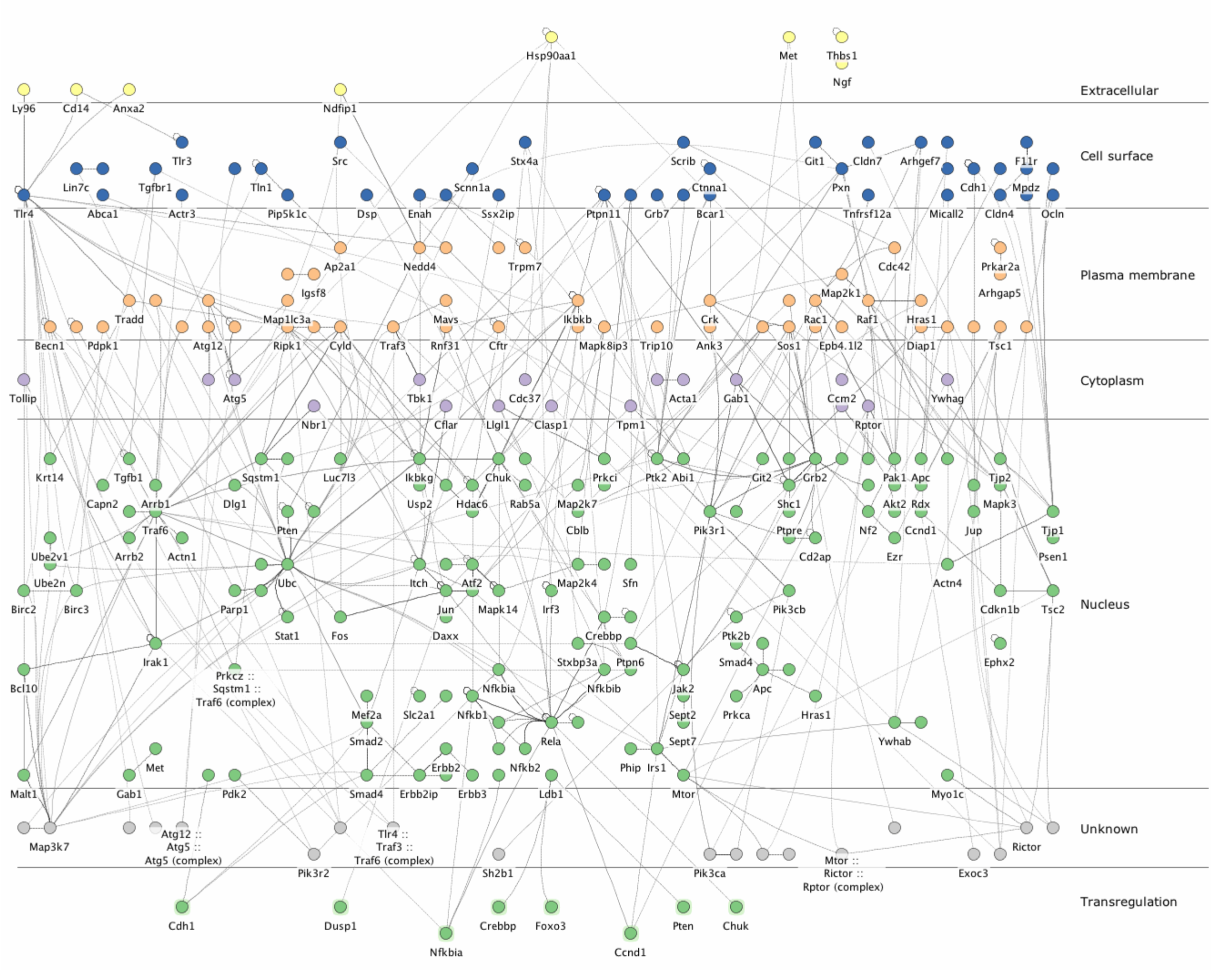

Cell Region-Based Rendering And Layout (Cerebral) [1] of an interactome model based on the LE-members associated with enrichments in the EM's transcriptional landscape during transition form pre- to subclinical SS in C57BL/6.NOD-*Aec1*Aec2 mice between 4 and 8 weeks of age (Figure 3 & 4). Mining of the experimental data was computed using InnateDB [2]. **Line opacity** = strength of experimental evidence; **node color** = subcellular localization (not curated).

Supplementary References

1. Barsky A, Gardy JL, Hancock RE, Munzner T: **Cerebral: a Cytoscape plugin for layout of and interaction with biological networks using subcellular localization annotation**. *Bioinformatics* 2007, **23**(8):1040-1042.
2. Lynn DJ, Winsor GL, Chan C, Richard N, Laird MR, Barsky A, Gardy JL, Roche FM, Chan TH, Shah N *et al*: **InnateDB: facilitating systems-level analyses of the mammalian innate immune response**. *Mol Syst Biol* 2008, **4**:218.
