# Supplementary material for: Transcriptional landscapes of emerging autoimmunity: transient aberrations in the targeted tissue’s extracellular milieu precede immune responses in Sjögren’s syndrome
Source: Arthritis Res Ther. 2013 Oct 31;15(5):R174. doi: 10.1186/ar4362 (PMC3978466; doi:10.1186/ar4362)

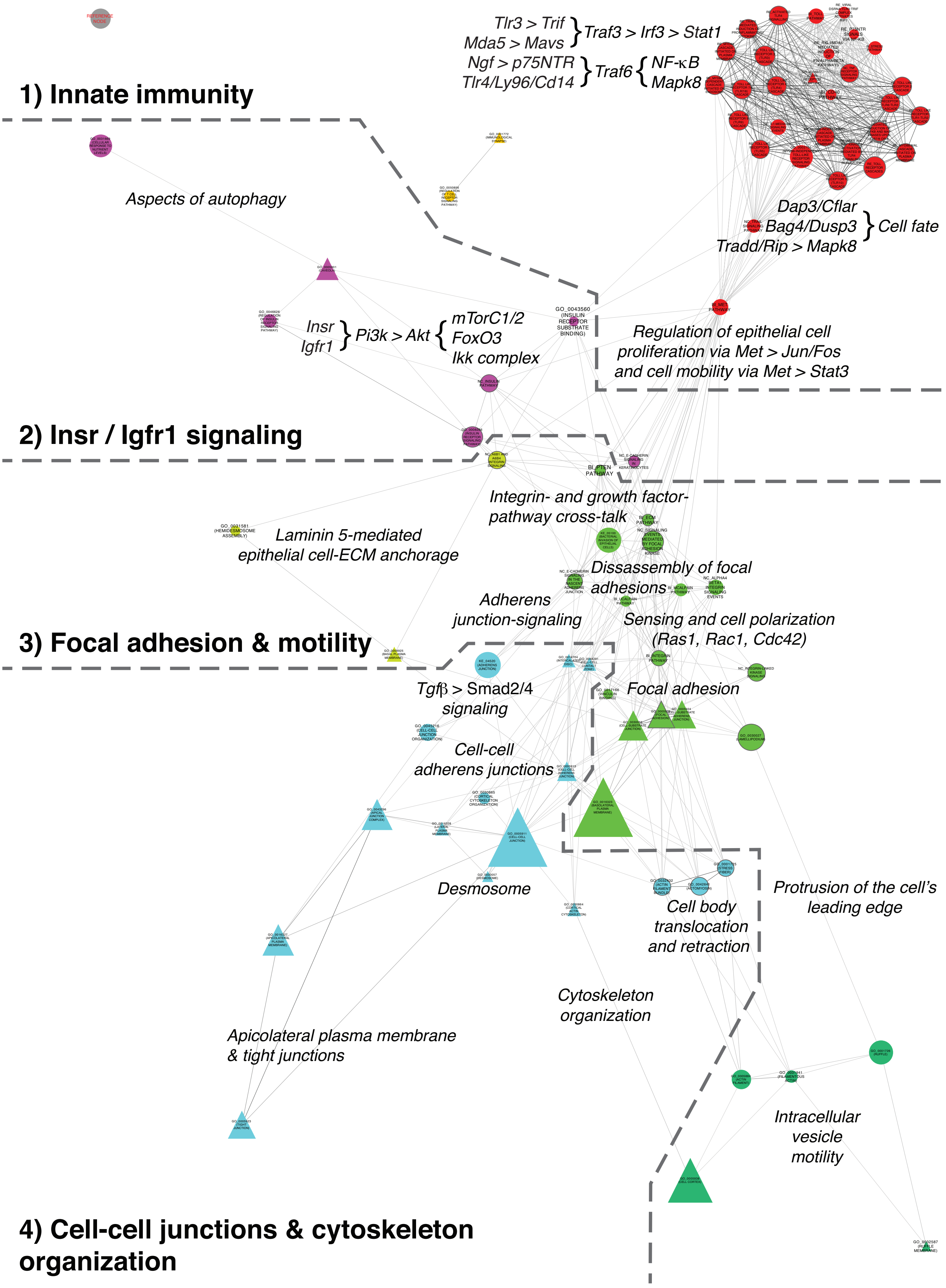
1) Innate immunity
Tlr3 > Trif
Mda5 > Mavs
Traf3 > Irf3 > Stat1
Ngf > p75NTR
Tlr4/Ly96/Cd14
Traf6
NF-κB
Mapk8
Aspects of autophagy
Dap3/Cflar
Bag4/Dusp3
Tradd/Rip > Mapk8
Cell fate
Insr
Igfr1
Pi3k > Akt
mTorC1/2
FoxO3
Ikk complex
Regulation of epithelial cell
proliferation via Met > Jun/Fos
and cell mobility via Met > Stat3
2) Insr / Igfr1 signaling
Integrin- and growth factor-
pathway cross-talk
Laminin 5-mediated
epithelial cell-ECM anchorage
Dissassembly of focal
adhesions
Adherens
junction-signaling
Sensing and cell polarization
(Ras1, Rac1, Cdc42)
3) Focal adhesion & motility
Tgfβ > Smad2/4
signaling
Focal adhesion
Cell-cell
adherens junctions
Desmosome
Protrusion of the cell's
leading edge
Cell body
translocation
and retraction
Cytoskeleton
organization
Apicolateral plasma membrane
& tight junctions
Intracellular
vesicle
motility
4) Cell-cell junctions & cytoskeleton
organization

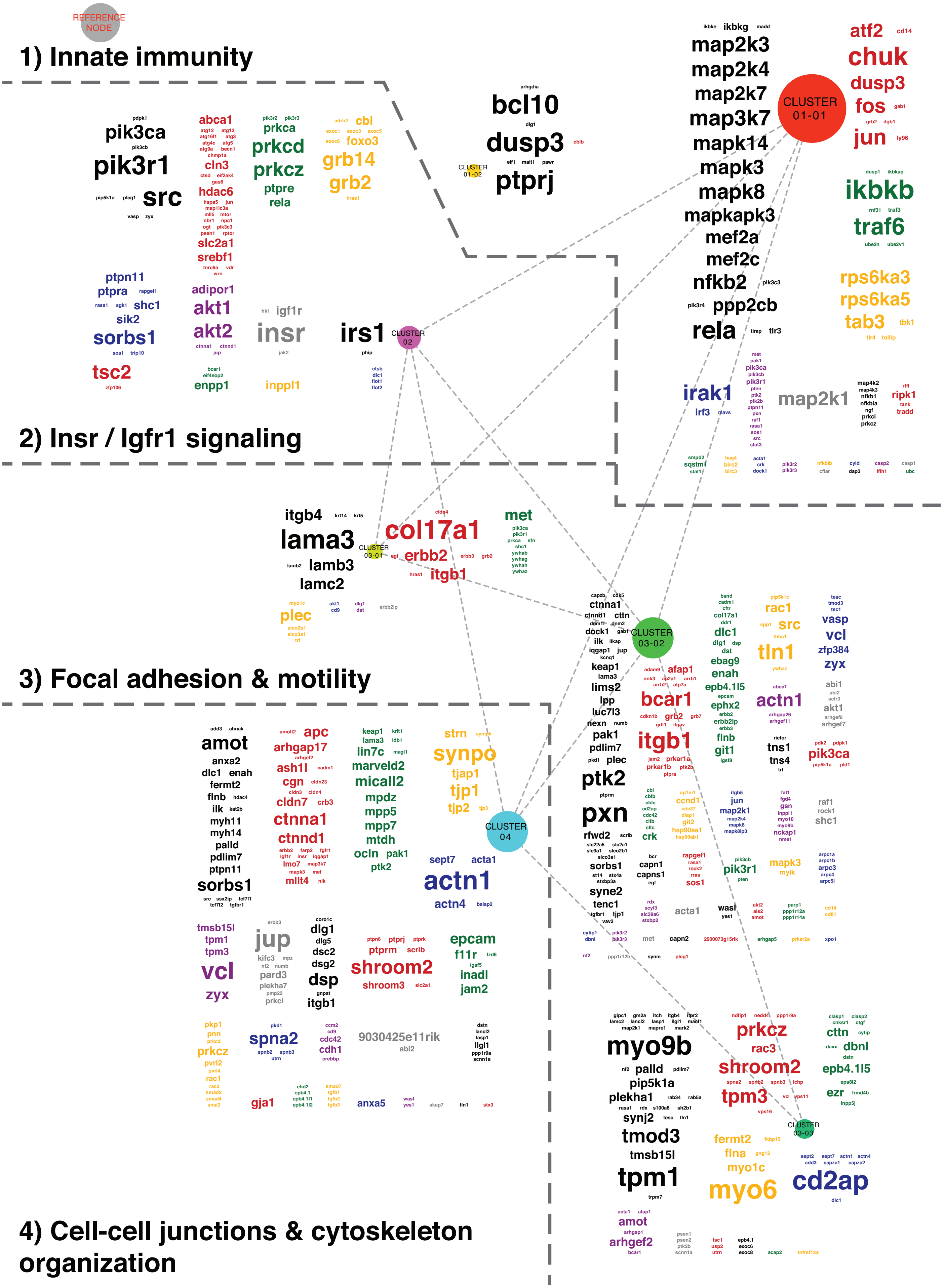

REFERENCE NODE
1) Innate immunity
2) Insr / Igfr1 signaling
3) Focal adhesion & motility
4) Cell-cell junctions & cytoskeleton organization
CLUSTER 01-01
CLUSTER 01-02
CLUSTER 02
CLUSTER 03-01
CLUSTER 03-02
CLUSTER 03-03
CLUSTER 04
pik3ca
pik3r1
src
abca1
cln3
hdac6
slc2a1
srebf1
prkca
prkcd
prkcz
ptpre
rela
cbl
foxo3
grb14
grb2
bcl10
dusp3
ptprj
ikbkg
map2k3
map2k4
map2k7
map3k7
mapk14
mapk3
mapk8
mapkapk3
mef2a
mef2c
nfkb2
ppp2cb
rela
atf2
chuk
dusp3
fos
jun
ikbkb
traf6
rps6ka3
rps6ka5
tab3
irak1
irf3
map2k1
ripk1
ptpn11
ptpra
shc1
sik2
sorbs1
tsc2
adipor1
akt1
akt2
enpp1
igf1r
insr
inppl1
irs1
itgb4
lama3
lamb3
lamc2
col17a1
erbb2
itgb1
met
plec
ctnna1
dock1
keap1
lims2
lpp
luc7l3
nexn
pak1
pdlim7
plec
ptk2
pxn
rfwd2
sorbs1
syne2
tenc1
rac1
src
tln1
vasp
vcl
zfp384
zyx
dlc1
ebag9
enah
epb4.1l5
ephx2
flnb
git1
afap1
bcar1
grb2
itgb1
actn1
akt1
tns1
tns4
pik3ca
ccnd1
crk
capn1
capns1
jun
map2k1
gsn
nckap1
pik3r1
mapk3
arpc3
raf1
shc1
acta1
wasl
amot
anxa2
dlc1
enah
fermt2
flnb
ilk
myh11
myh14
palld
pdlim7
ptpn11
sorbs1
apc
arhgap17
ash1l
cgn
cldn7
ctnna1
ctnnd1
lmo7
mllt4
keap1
lama3
lin7c
marveld2
micall2
mpdz
mpp5
mpp7
mtdh
ocln
pak1
ptk2
strn
synpo
tjap1
tjp1
tjp2
sept7
acta1
actn1
actn4
tmsb15l
tpm1
tpm3
vcl
zyx
jup
kifc3
pard3
plekha7
prkci
dlg1
dlg5
dsc2
dsg2
dsp
itgb1
ptprm
scrib
shroom2
shroom3
epcam
f11r
inadl
jam2
pkp1
pnn
prkcz
pvrl2
rac1
gja1
spna2
ccm2
cdc42
cdh1
9030425e11rik
abi2
anxa5
myo9b
palld
pip5k1a
plekha1
synj2
tmod3
tmsb15l
tpm1
prkcz
rac3
shroom2
tpm3
fermt2
flna
myo1c
myo6
cttn
dbnl
epb4.1l5
ezr
cd2ap
amot
arhgef2

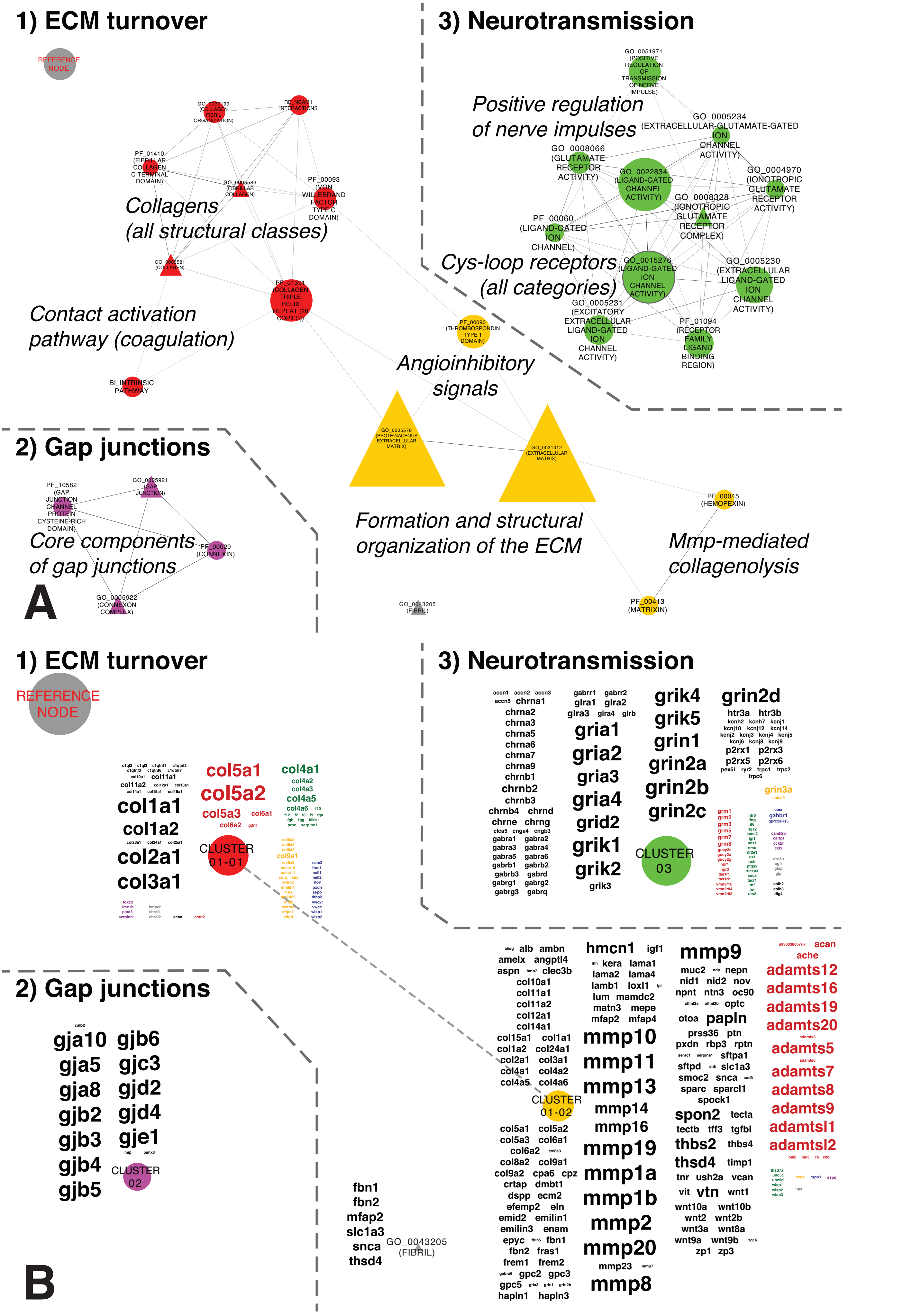
1) ECM turnover
REFERENCE NODE
Collagens (all structural classes)
Contact activation pathway (coagulation)
3) Neurotransmission
Positive regulation of nerve impulses
Cys-loop receptors (all categories)
Angioinhibitory signals
2) Gap junctions
Core components of gap junctions
Formation and structural organization of the ECM
Mmp-mediated collagenolysis
A
1) ECM turnover
REFERENCE NODE
CLUSTER 01-01
3) Neurotransmission
CLUSTER 03
2) Gap junctions
CLUSTER 02
CLUSTER 01-02
GO_0043205 (FIBRIL)
B

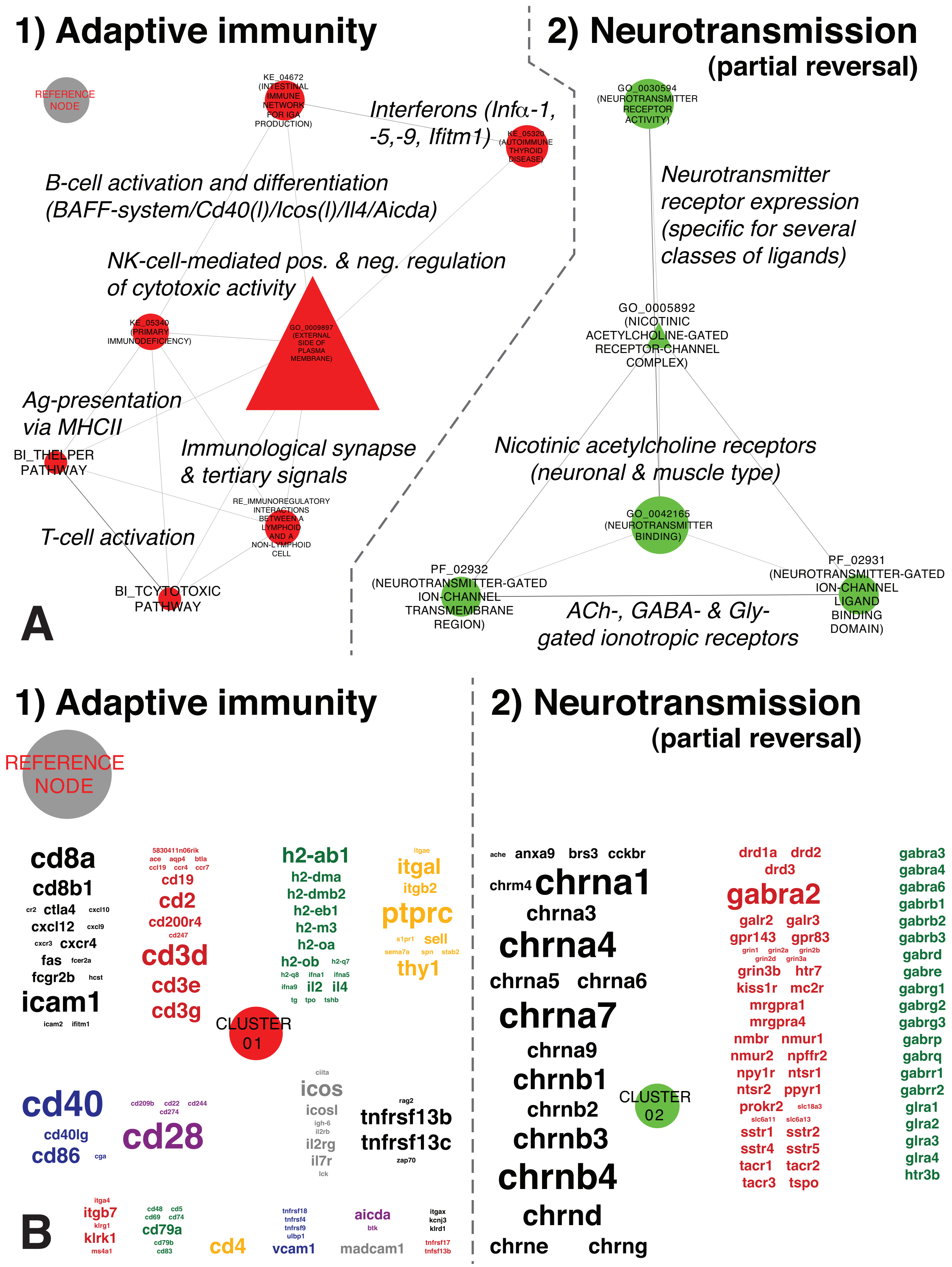

1) Adaptive immunity
REFERENCE NODE
KE_04672 (INTESTINAL IMMUNE NETWORK FOR IGA PRODUCTION)
Interferons (Infα-1, -5,-9, Ifitm1)
KE_05320 (AUTOIMMUNE THYROID DISEASE)
B-cell activation and differentiation (BAFF-system/Cd40(l)/Icos(l)/Il4/Aicda)
NK-cell-mediated pos. & neg. regulation of cytotoxic activity
KE_05340 (PRIMARY IMMUNODEFICIENCY)
GO_0009897 (EXTERNAL SIDE OF PLASMA MEMBRANE)
Ag-presentation via MHCII
BI_THELPER PATHWAY
Immunological synapse & tertiary signals
RE_IMMUNOREGULATORY INTERACTIONS BETWEEN A LYMPHOID AND A NON-LYMPHOID CELL
T-cell activation
BI_TCYTOTOXIC PATHWAY
A
2) Neurotransmission (partial reversal)
GO_0030594 (NEUROTRANSMITTER RECEPTOR ACTIVITY)
Neurotransmitter receptor expression (specific for several classes of ligands)
GO_0005892 (NICOTINIC ACETYLCHOLINE-GATED RECEPTOR-CHANNEL COMPLEX)
Nicotinic acetylcholine receptors (neuronal & muscle type)
GO_0042165 (NEUROTRANSMITTER BINDING)
PF_02932 (NEUROTRANSMITTER-GATED ION-CHANNEL TRANSMEMBRANE REGION)
PF_02931 (NEUROTRANSMITTER-GATED ION-CHANNEL LIGAND BINDING DOMAIN)
ACh-, GABA- & Gly-gated ionotropic receptors
1) Adaptive immunity
REFERENCE NODE
cd8a cd8b1 cr2 ctla4 cxcl10 cxcl12 cxcl9 cxcr3 cxcr4 fas fcer2a fcgr2b hcst icam1 icam2 ifitm1
5830411n06rik ace aqp4 btla ccl19 ccr4 ccr7 cd19 cd2 cd200r4 cd247 cd3d cd3e cd3g
h2-ab1 h2-dma h2-dmb2 h2-eb1 h2-m3 h2-oa h2-ob h2-q7 h2-q8 ifna1 ifna5 ifna9 il2 il4 tg tpo tshb
itgae itgal itgb2 ptprc s1pr1 sell sema7a spn stab2 thy1
CLUSTER 01
cd40 cd40lg cd86 cga
cd209b cd22 cd244 cd274 cd28
ciita icos icosl igh-6 il2rb il2rg il7r lck
rag2 tnfrsf13b tnfrsf13c zap70
B
itga4 itgb7 klrg1 klrk1 ms4a1
cd48 cd5 cd69 cd74 cd79a cd79b cd83
cd4
tnfrsf18 tnfrsf4 tnfrsf9 ulbp1 vcam1
aicda btk madcam1
itgax kcnj3 klrd1 tnfrsf17 tnfsf13b
2) Neurotransmission (partial reversal)
ache anxa9 brs3 cckbr chrm4 chrna1 chrna3 chrna4 chrna5 chrna6 chrna7 chrna9 chrnb1 chrnb2 chrnb3 chrnb4 chrnd chrne chrng
CLUSTER 02
drd1a drd2 drd3 gabra2 galr2 galr3 gpr143 gpr83 grin1 grin2a grin2b grin2d grin3a grin3b htr7 kiss1r mc2r mrgpra1 mrgpra4 nmbr nmur1 nmur2 npffr2 npy1r ntsr1 ntsr2 ppyr1 prokr2 slc18a3 slc6a11 slc6a13 sstr1 sstr2 sstr4 sstr5 tacr1 tacr2 tacr3 tspo
gabra3 gabra4 gabra6 gabrb1 gabrb2 gabrb3 gabrd gabre gabrg1 gabrg2 gabrg3 gabrp gabrq gabrr1 gabrr2 glra1 glra2 glra3 glra4 htr3b

1) Focal adhesion & cell-cell junctions (partial reversal)

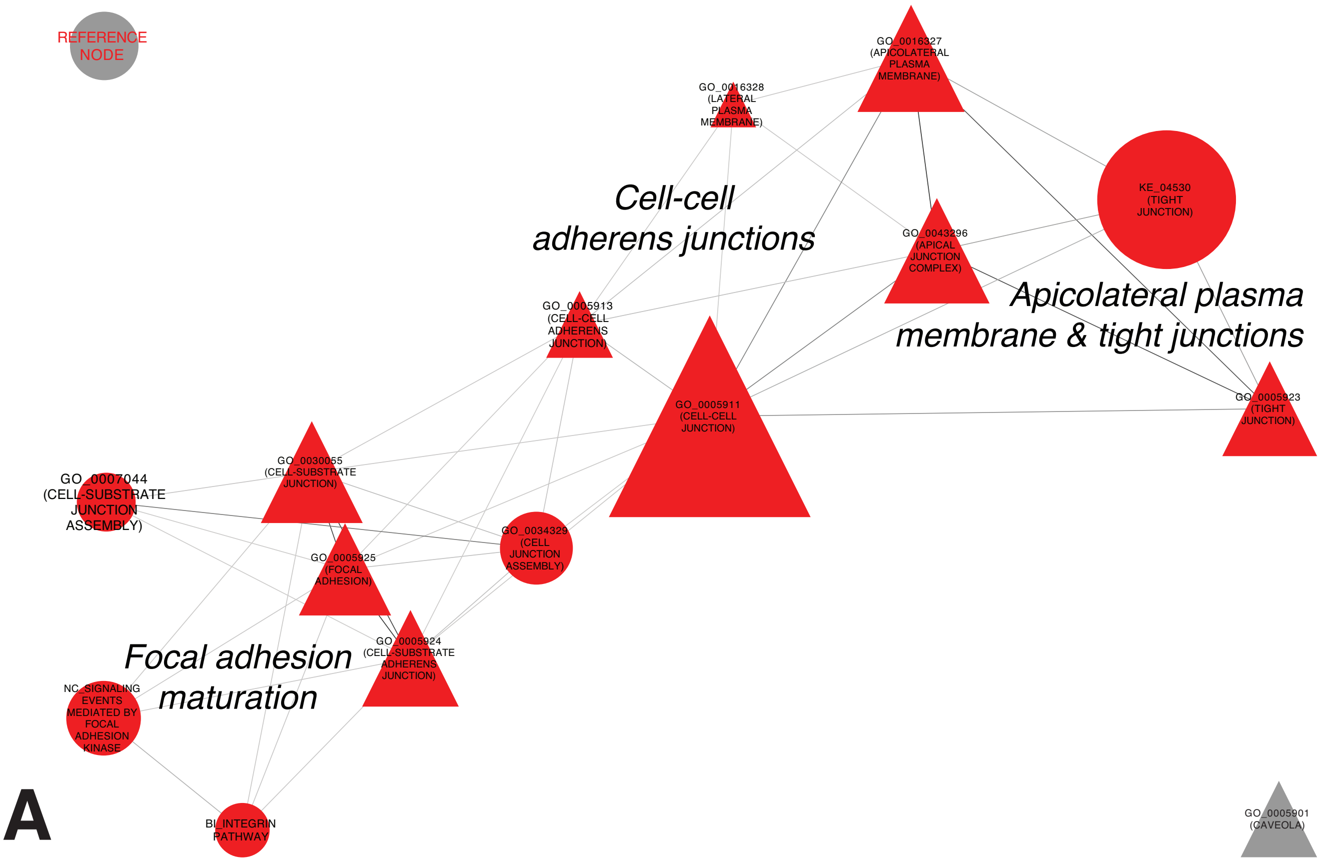

1) Focal adhesion & cell-cell junctions (partial reversal)

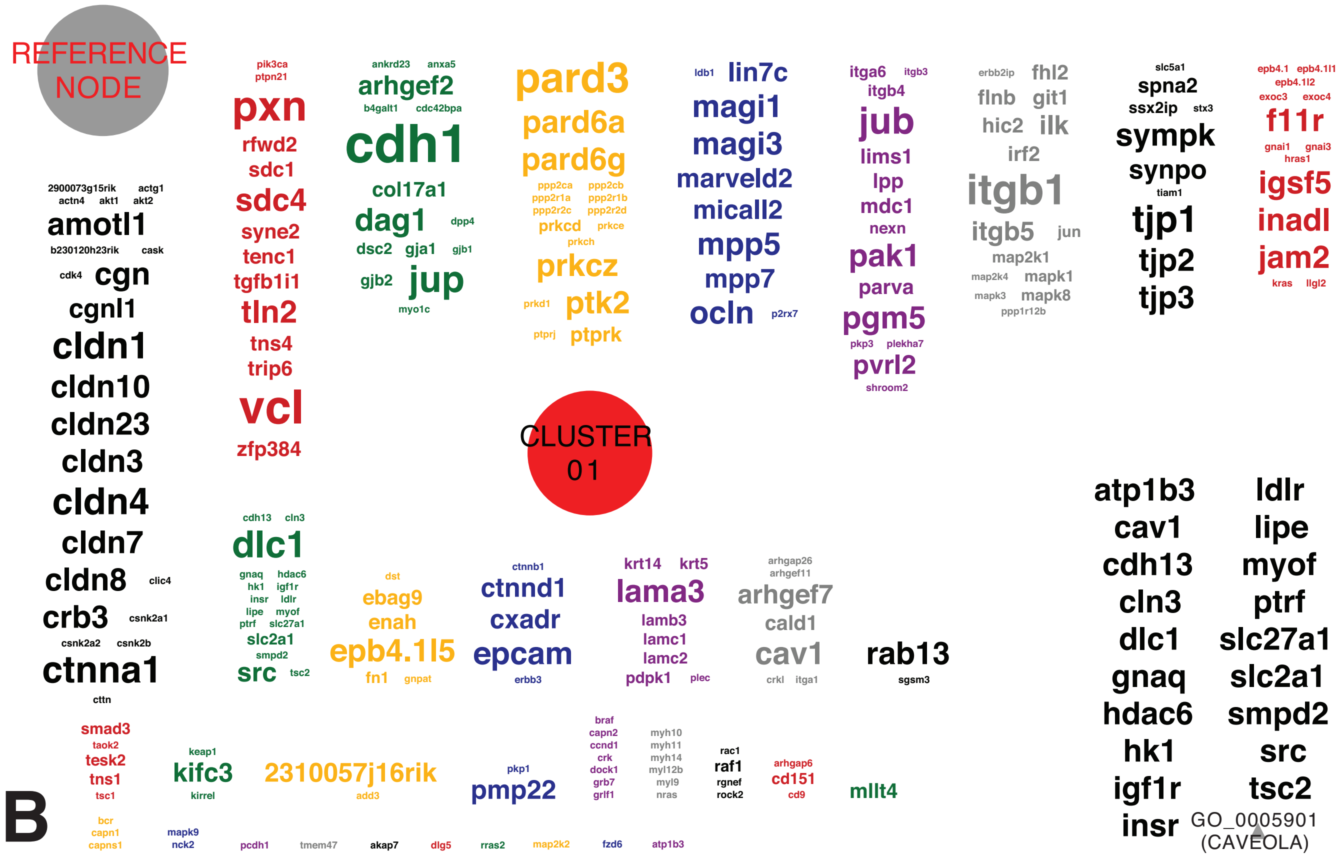

Supplement: Additional file 3 — The networks displayed in Figures 3-7 as infinitely scalable and electronically searchable vector graphics. [file ar4362-S3.pdf]
